# Calcium channel ITPR2 and mitochondria–ER contacts promote cellular senescence and aging

**DOI:** 10.1038/s41467-021-20993-z

**Published:** 2021-02-01

**Authors:** Dorian V. Ziegler, David Vindrieux, Delphine Goehrig, Sara Jaber, Guillaume Collin, Audrey Griveau, Clotilde Wiel, Nadia Bendridi, Sophia Djebali, Valerio Farfariello, Natacha Prevarskaya, Léa Payen, Jacqueline Marvel, Sébastien Aubert, Jean-Michel Flaman, Jennifer Rieusset, Nadine Martin, David Bernard

**Affiliations:** 1 Centre de Recherche en Cancérologie de Lyon, Inserm U1052, CNRS UMR 5286, Centre Léon Bérard, Université de Lyon, Lyon, France; 2 CarMeN Laboratory, INSERM UMR-1060, Lyon 1 University, INRA U1397, F-69921 Oullins, France; 3 Centre International de Recherche en Infectiologie, Inserm U1111, CNRS UMR5308, École Normale Supérieure de Lyon, Université de Lyon, Université Claude Bernard Lyon 1, Lyon, France; 4 INSERM U1003, Laboratoire d’Excellence, Canaux Ioniques d’Intérêt Thérapeutique, Équipe Labellisée Par la Ligue Nationale Contre le Cancer, SIRIC ONCOLille, Université des Sciences et Technologies de Lille, Villeneuve d’Ascq, France; 5 Institut de Pathologie, Centre de Biologie Pathologie, CHRU de Lille, Faculté de Médecine, Université de Lille, Lille Cedex, France

**Keywords:** Mechanisms of disease, Senescence

## Abstract

Cellular senescence is induced by stresses and results in a stable proliferation arrest accompanied by a pro-inflammatory secretome. Senescent cells accumulate during aging, promoting various age-related pathologies and limiting lifespan. The endoplasmic reticulum (ER) inositol 1,4,5-trisphosphate receptor, type 2 (ITPR2) calcium-release channel and calcium fluxes from the ER to the mitochondria are drivers of senescence in human cells. Here we show that *Itpr2* knockout (KO) mice display improved aging such as increased lifespan, a better response to metabolic stress, less immunosenescence, as well as less liver steatosis and fibrosis. Cellular senescence, which is known to promote these alterations, is decreased in *Itpr2* KO mice and *Itpr2* KO embryo-derived cells. Interestingly, ablation of ITPR2 in vivo and in vitro decreases the number of contacts between the mitochondria and the ER and their forced contacts induce premature senescence. These findings shed light on the role of contacts and facilitated exchanges between the ER and the mitochondria through ITPR2 in regulating senescence and aging.

## Abstract (web summary)

Contacts between mitochondria and endoplasmatic reticulum (ER), and the transfer of calcium between them, have an important role in the regulation of cellular phenotypes, including senescence. Here the authors show that ITPR2 deficient mice display improved aging, associated with a decreased number of contacts between the mitochondria and the ER.

## Introduction

Cellular senescence is an important process regulating different pathophysiological processes from embryonic development to aging. In particular, it promotes various age-related diseases and shortens the lifespan^1–3^. Cellular senescence is characterized by a stable cell cycle arrest and a pro-inflammatory senescent-associated secretory program (SASP), both involved in the pathophysiological effects of senescent cells^4,5^. Although downstream factors, such as p16, directly blocking cell cycle or activating the SASP are largely described, the upstream molecular and sub-cellular mechanisms controlling these factors are largely unknown. The network and activity of mitochondria are dysregulated during cellular senescence, though the characterization and cause of these alterations are largely unknown^6^.

Calcium critically regulates many cellular and molecular processes including but not limited to secretion, autophagy, migration, proliferation and cell death^7^. More recently calcium has been shown to be regulated during cellular senescence and to impact its outcome^8,9^. We and others have recently shown that transfer of calcium from the endoplasmic reticulum (ER) through inositol 1,4,5-trisphosphate receptor (ITPR or IP_3_R) ER channels to the mitochondria and its subsequent mitochondrial accumulation leads to cellular senescence in normal human cells^9–12^. Contact sites between mitochondria and ER, also called mitochondria-ER contacts (MERCs) or mitochondria-associated ER membranes (MAMs), have emerged as hotspots for calcium transfer and signaling^13–16^. ITPR2 can be part of MERC sites, coordinating among others calcium transfer^16^. Potential role of these MERC sites in cellular senescence is currently unknown.

Here, we show that ITPR2 promotes some age-associated hallmarks and aging in mice. This is associated with increased cellular senescence and MERCs in mice. Accordingly, *Itpr2* KO mouse embryonic fibroblasts (MEFs) display less senescence, reduced MERCs and limited mitochondrial calcium. Furthermore, forcing MERCs by using a synthetic linker induces premature senescence. Taken together, these results support that ITPR2 and increased MERCs are important regulators of cellular senescence and aging.

## Results

### *Itpr2* knockout increases lifespan and limits age-related phenotypes in mice

**Fig. 1:**
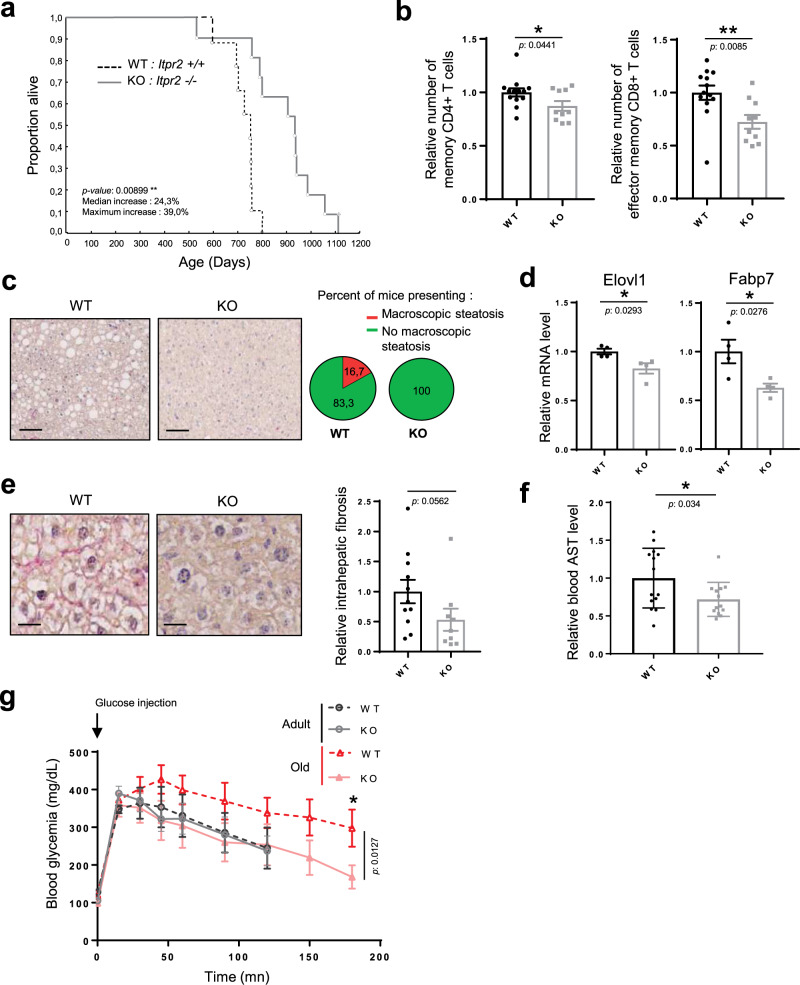
*Itpr2* knockout increases lifespan and limits age-related phenotypes in mice. **a** Survival curves of *Itpr2* WT (*n* = 9) or KO (*n* = 11) C57BL/6 female mice. Log-rank test. **b** Immunophenotyping of the spleens of 23-month-old mice displaying a relative number of memory (CD44high) CD4+ and CD8+ T-cells in *Itpr2* WT (*n* = 13) and KO (*n* = 10) female and male mice. Mean ± SEM. Unpaired two-tailed Student *t*-test. **c** Micrographs of liver sections stained with Sirius Red of 23-month-old male and female *Itpr2* WT (*n* = 12) and KO (*n* = 10) mice. Scale bar: 25 µm. Quantification of the percentage of mice presenting macroscopic steatosis. **d** mRNA level extracted from microarray data in the livers of 23-month-old WT (*n* = 4) and KO (*n* = 4) female mice. Mean ± SEM. Unpaired two-tailed Student *t*-test. **e** Micrographs of liver sections stained with Sirius Red. Scale bar: 10 µm. Quantification of relative intrahepatic collagen fibers according to Sirius Red staining in 23-month-old male and female *Itpr2* WT (*n* = 12) and KO (*n* = 10) mice. Mean ± SEM. Two-tailed Mann–Whitney *U* Test. **f** Quantification of relative blood AST level of 23-month-old *Itpr2* WT (*n* = 14) and KO (*n* = 13) female and male mice. Mean ± SEM. Unpaired two-tailed Student *t*-test. **g** Intraperitoneal glucose-tolerance test of adult (20-month-old) and old (26-month-old) *Itpr2* WT (*n* = 6) and KO (*n* = 8) male mice. Mean ± SEM. Unpaired two-tailed Mann–Whitney *U* Test. *p < 0.05; **p < 0.01; ***p < 0.001.

In order to investigate the importance of ITPR2 in physiological aging, we studied and monitored cohorts of *Itpr2* knockout mice (KO)^17^ up to their death. Remarkably, *Itpr2* KO enhanced the median and maximum lifespan of female mice by 23% and 39%, respectively (Fig. 1a). Loss of *Itpr2* did not affect the survival of male mice (Supplementary Fig. 1a). The differences in lifespan between males and females are well described. Indeed, many studies report striking gender-associated lifespan differences following a modification in regimen or gene expression, though the mechanisms underlying such differences remain unclear^18–21^. According to our results, WT males lived longer than WT females as already reported^22^, suggesting that *Itpr2* could contribute to the intrinsic lifespan difference between male and female mice.

Aging is a complex systemic process involving numerous vital organs. Several aging-related features were monitored: immunosenescence designated as immune aging^23^, liver alterations, response to a metabolic challenge, bone density and tumor lesions. Immunosenescence, essentially characterized by the exhaustion of naive T cells and accumulation of memory T cells, was examined by quantifying the differences between these two populations^24^ in the spleens of 23-month old WT and *Itpr2* KO mice. *Itpr2* KO mice displayed a decreased number of both memory CD4+ T cells and effector memory CD8+ T cells compared to control littermates (Fig. 1b). This decrease was associated with an increased proportion of both naive CD4+ and CD8+ T cells, and an overall increase in naive/memory T cells (Supplementary Fig. 1b), strongly supporting that ITPR2 promotes immunosenescence in aged mice.

Since *Itpr2* is highly expressed in the liver^25^, we examined the structure and function of the liver in aged mice. Compared to WT mice, the livers of old *Itpr2* KO mice displayed no marks of macroscopic steatosis (Fig. 1c) and fewer lipid droplets (Supplementary Fig. 1c). Accumulation of lipid is a key feature of liver steatosis^26^, mainly through increased triacylglycerol (TAG) synthesis. Whole-genome transcriptome analysis revealed that loss of *Itpr2* reduced the cellular lipid metabolic Gene Ontology signature and the fatty acid metabolism Gene set in the liver of old mice (Supplementary Fig. 1d, e). More specifically, mRNA levels of the fatty acid biosynthesis enzymes Elovl1 (elongation of very long-chain fatty acids protein 1) and Fabp7 (free fatty acid-binding protein 7) were significantly reduced in the liver of old *Itpr2* KO female mice (Fig. 1d). Moreover, *Itpr2* KO mice displayed decreased liver fibrosis (Fig. 1e) and presented a decreased blood aspartate aminotransferase (AST) level (Fig. 1f), a marker of damaged liver^27^.

We next investigated the ability of old WT and *Itpr2* KO mice to respond to a metabolic challenge, since during aging, liver dysfunction contributes to an altered response^28^. Although no difference between the two genotypes was observed in the regulation of glycaemia during a glucose tolerance test in 20-month-old mice, 26-month-old WT male mice had lost their ability to regulate their glycaemia, whereas KO littermates responded normally to glucose injection (Fig. 1g), suggesting that *Itpr2* KO mice were protected from age-induced glucose intolerance. Furthermore, *Itpr2* KO resulted in the abolition of age-related increased basal blood glycaemia (Supplementary Fig. 1f). This parameter could not be assessed in females as most of the females were already dead at this age (Fig. 1a). Besides, no significant differences were observed between old WT and *Itpr2* KO mice for the following parameters: bone mineral density and content (Supplementary Fig. 1g), weight (Supplementary Fig. 1h), and tumor lesions (Supplementary Fig. 1i).

Together, these results support that ITPR2 promotes some alterations associated with physiological aging.

### Loss of *Itpr2* reduces cellular senescence in mice and their derived cells

**Fig. 2:**
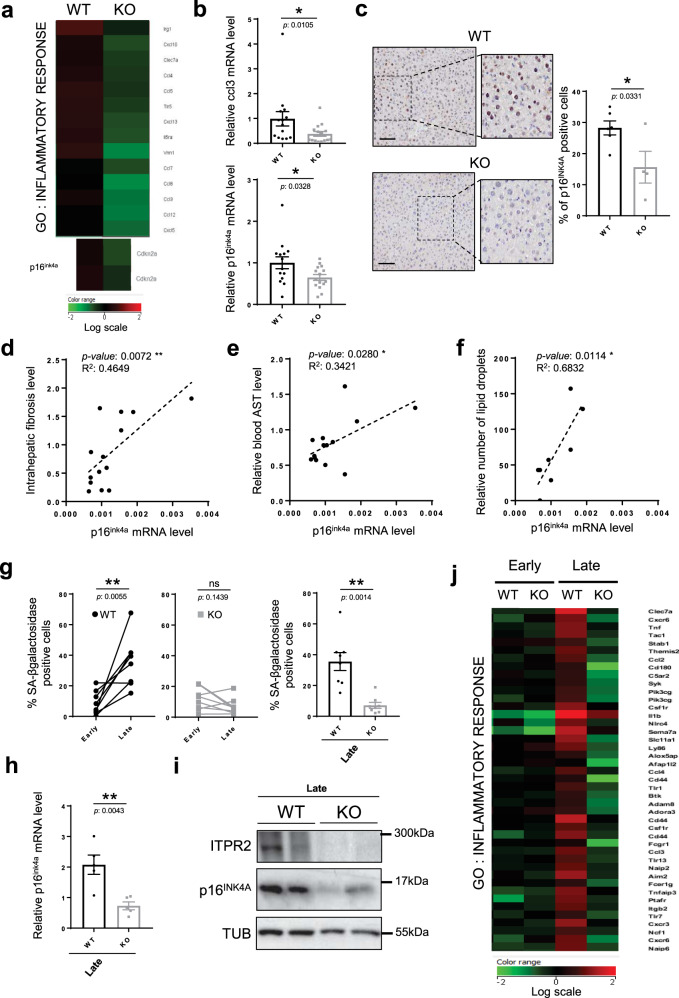
Loss of *Itpr2* in mice reduces key features of cellular senescence. **a** Inflammatory response gene ontology obtained from the genes downregulated in the livers of 23-month-old *Itpr2* WT (*n* = 4) and KO (*n* = 4) female mice, according to transcriptomic microarray analyses, corrected *p*-value = 0.019. **b** Relative ccl3 and p16^ink4a^ mRNA levels in liver of 23-month-old *Itpr2* WT (*n* = 14) and KO (*n* = 15) female and male mice. Mean ± SEM. Unpaired two-tailed Mann–Whitney *U* Test. **c** Micrographs of liver sections and quantification of p16^INK4A^-positive cells, stained by immunohistochemistry (IHC) in the livers of 23-month-old mice *Itpr2* WT (*n* = 4) and KO (*n* = 4) female mice. Mean ± SEM. Unpaired two-tailed Student *t*-test (*p = 0.0331). **d**–**e** Linear regression analyses between intrahepatic fibrosis level or blood AST level and p16^ink4a^ mRNA levels in the livers of 23-month-old female and male mice (*n* = 14). Two-tailed Spearman Rank Correlation test. **f** Linear regression analysis between the number of lipid droplets and p16^ink4a^ mRNA levels in the liver of 23-month-old female mice (*n* = 7). Two-tailed Spearman Rank Correlation test. **g** Quantification of SA-ß-galactosidase-positive cells in *Itpr2* WT (*n* = 8) and KO (*n* = 7) MEFs at early and late passage. Mean ± SEM. Early vs. Late: Paired two-tailed Student *t*-test (ns: non-significant). Late WT vs. Late KO: Unpaired two-tailed Welch’s *t*-test. **h** p16^ink4a^ mRNA level in WT (*n* = 5) and KO (*n* = 5) MEFs at late passage. Mean ± SEM. Unpaired two-tailed Welch’s *t*-test. **i** p16^INK4A^, Itpr2 and tubulin protein levels in *Itpr2* WT and KO MEFs at late passage. **j** Inflammatory response gene ontology extracted from microarray of *Itpr2* WT (*n* = 3) and KO (*n* = 3) MEFs at early and late passage, corrected *p*-value = 2.292^−12^. *p < 0.05; **p < 0.01; ***p < 0.001; ns non-significant.

As increased cellular senescence is known to promote all of the age-related alterations described above^4,5^ and as the ITPR2 calcium channel is a positive regulator of cellular senescence in human cells^10^, we wondered whether loss of *Itpr2* decreased cellular senescence in mice and in mice-derived cells. Transcriptomic analyses revealed that liver from 23-month-old *Itpr2* KO mice presented fewer markers of cellular senescence, as evidenced by a decrease in the inflammatory response signature, a signature that can be related to pro-inflammatory SASP^29^ and includes ccl3, ccl4, ccl12, cxcl5 and cxcl10, and p16^ink4a^ mRNA levels (Fig. 2a and Supplementary Fig. 2a). These results were confirmed in a larger group of mice by RT-qPCR on ccl3 and p16^ink4a^ (Fig. 2b) and by the immunohistochemical analysis of the p16^INK4A^ protein (Fig. 2c).

Reminiscent of *Itpr2* loss extending the lifespan of female animals only, ccl3 and p16^ink4a^ mRNA levels were markedly reduced in the liver of old *Itpr2* KO females compared to livers of old WT females. A similar but weaker trend towards a reduction in ccl3 and p16^ink4a^ expression was observed in the liver of old *Itrp2* KO males compared to the liver of old WT males (Supplementary Fig. 2b). In addition, p16^ink4a^ mRNA levels were two-fold higher in the liver of old WT females compared to WT males, consistent with the difference in lifespan observed above between the sexes (Fig. 1a and Supplementary Fig. 1a). In line with a role for cellular senescence in promoting liver aging and dysfunction^30–32^, intrahepatic fibrosis level as well as blood AST levels and lipid droplets quantity were positively correlated with the p16^ink4a^ senescence marker in the liver of old mice (Fig. 2d–f).

To further confirm a link between the loss of *Itpr2* and decreased levels of senescence, beyond the liver, we explored the replicative potential of *Itpr2* KO- and WT-derived MEFs. Late passage *Itpr2* KO MEFs showed a decreased level of cellular senescence, as illustrated by a decrease in the level of several cellular senescence markers, namely the senescence associated-β-galactosidase activity (SA-β-Gal) (Fig. 2g and Supplementary Fig. 2c), cell proliferation (Supplementary Fig. 2d), p16^ink4a^ mRNA (Fig. 2h) and protein (Fig. 2i) levels and finally a reversion of the enhanced inflammatory response as evidenced by transcriptomic data (Fig. 2j and Supplementary Fig. 2e).

Because ITPR2 can mediate calcium efflux from the ER to the mitochondria and mitochondrial calcium can mediate senescence^10,12^, we determined calcium levels in the late passage *Itpr2* KO and WT MEFs, using ratiometric genetic reporters. *Itpr2* KO MEFs displayed higher ER calcium levels and lower mitochondrial calcium levels when compared to the WT late passage MEFs (Supplementary Fig. 2f, g), as well as decreased mitochondrial depolarization and mitochondrial ROS production (Supplementary Fig. 2h, i). These results strongly suggested that old *Itpr2* KO MEFs, which undergo less senescence, have moderated calcium fluxes from the ER to mitochondria and attenuated mitochondrial dysfunction.

In conclusion, these results support that loss of *Itpr2* reduces the level of cellular senescence, limits ER-mitochondrial calcium fluxes, and is correlated with an improvement in the structure and function of the liver of old mice.

### Loss of *Itpr2* diminishes MERCs

ITPR2 can be part of and promotes the MERCs^16,33^, which are hotspots for calcium exchange between the ER and the mitochondria^15,34^. Moreover, ITPR2 promotes cellular senescence in human cells by favoring calcium transfer to the mitochondria^10^, and we observed here that late passage senescence-resistant *Itpr2* KO MEFs showed decreased levels of mitochondrial calcium (Supplementary Fig. 2g). We thus evaluated whether cellular senescence regulated by ITPR2 could involve MERCs.

**Fig. 3:**
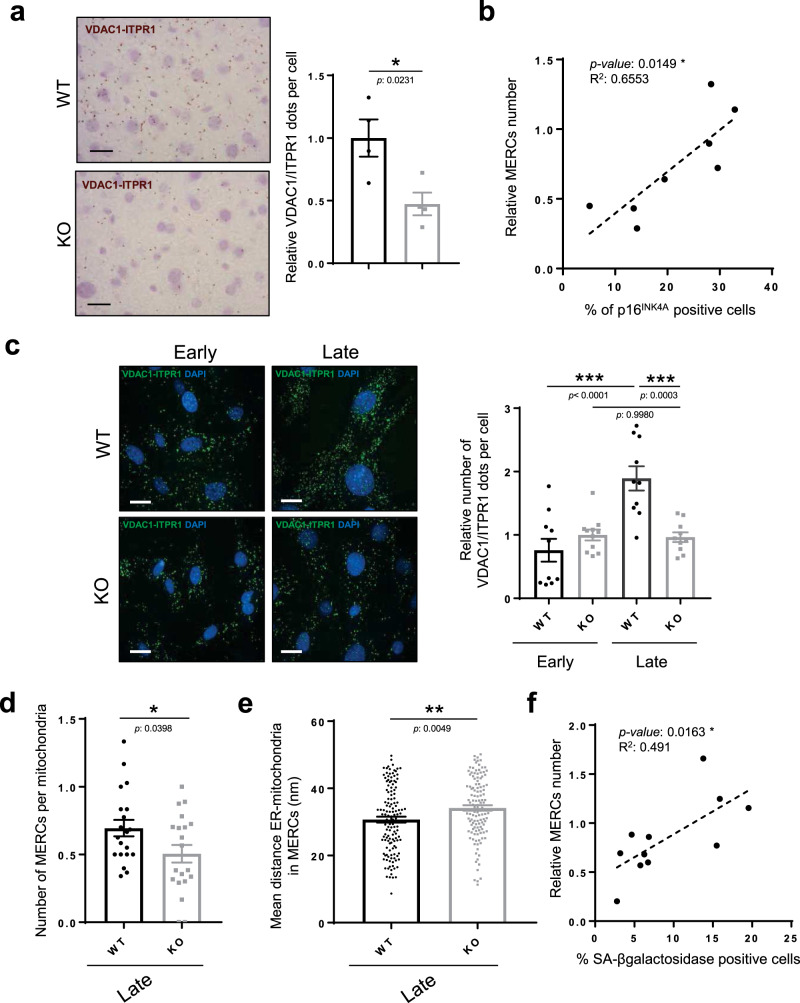
Loss of *Itpr2* diminishes contacts between mitochondria and the ER. **a** Proximity ligation assay (PLA) using VDAC1 (outer mitochondrial membrane) and ITPR1 (ER membrane) antibodies in the livers of 23-month-old *Itpr2* WT (*n* = 4) and KO (*n* = 4) female mice. Dots are formed when the distance between ITPR1 and VDAC1 is below 50 nm. Representative pictures (scale bar: 5 µm) and quantification shown as mean ± SEM. Unpaired two-tailed Student *t*-test. **b** Linear regression analysis between the number of MERCs and the percentage of p16^INK4A^positive cells in the liver of 23-month-old *Itpr2* WT (*n* = 4) and KO (*n* = 4) female mice. Two-tailed Spearman Rank Correlation test. **c** PLA using Vdac1 and Itpr1 antibodies in early and late passage *Itpr2* WT and KO MEFs. Representative pictures (scale bar: 5 µm) and mean ± SEM of *n* = 10 fields, examined over *n* = 4 independent experiments. Two-way ANOVA. Tukey’s multiple comparisons test. **d** Quantification of the number of MERCs normalized to mitochondria number in late passage *Itpr2* WT and KO MEFs. MERCs are defined by a distance <50 nm between ER and OMM membranes, evaluated by transmission electron microscopy. Mean ± SEM of *n* = 20 cells. Unpaired two-tailed Student *t*-test. **e** Mean ER-mitochondria distance in MERCs in late passage *Itpr2* WT and KO MEFs, based on electron microscopy study. Mean ± SEM of 20 cells, *n* representing individual MERCs of *Itpr2* WT (*n* = 146) and KO (*n* = 138) MEFs examined over *n* = 3 independent biological replicates. Two-tailed Mann–Whitney *U* Test. **f** Linear regression analysis between the number of MERCs and percentage of SA-ß-galactosidase positive cells at different passages of *Itpr2* WT (*n* = 4) and KO (*n* = 4) MEFs. Two-tailed Spearman Rank Correlation test. *p < 0.05; **p < 0.01; ***p < 0.001.

Assessment of the number of MERCs was performed by examining close proximity between ER, using ITPR1, and mitochondria, using VDAC1, both proteins being components of MERCs, as previously described and validated in the liver of mice using proximity ligation assay (PLA)^35^. The number of MERCs was 2-fold lower in the liver of old *Itpr2* KO mice compared to WT littermates (Fig. 3a and Supplementary Fig. 3a) and these changes were not due to changes in ITPR1 and VDAC1 levels (Supplementary Fig. 3b, c). The number of MERCs was also correlated with the level of the senescence marker p16^INK4A^ (Fig. 3b and Supplementary Fig. 3d). Remarkably the number of MERCs in mice was positively correlated with the relative level of intrahepatic fibrosis (Supplementary Fig. 3e). Further supporting a link between ITPR2 levels, senescence and the number of MERCs, late passage *Itpr2* KO MEFs displayed a decrease in the number of MERCs compared to old and senescent WT MEFs, according to both PLAs between ITPR1 and VDAC1 (Fig. 3c), without any change in ITPR1 and VDAC1 levels (Supplementary Fig. 3f, g), and transmission electronic microscopy (TEM) (Fig. 3d and Supplementary Fig. 3h). Beyond the number of MERCs, the distance between the ER and the mitochondria in MERCs increased in late passage *Itpr2* KO MEFs (Fig. 3e and Supplementary Fig. 3i). Perimeter and number of mitochondria were not decreased in old *Itpr2* KO MEFs (Supplementary Fig. 3j), excluding this bias in the analysis of MERCs. As observed in vivo, the number of MERCs was also positively correlated with the senescence marker SA-β-Gal in MEFs (Fig. 3f), substantiating once again a link between MERCs and cellular senescence.

Hence, these results support that fewer and more relaxed MERCs in *Itrp2* KO mice and their derived MEFs are correlated with lower levels of cellular senescence.

### Increasing MERCs induces premature senescence

**Fig. 4:**
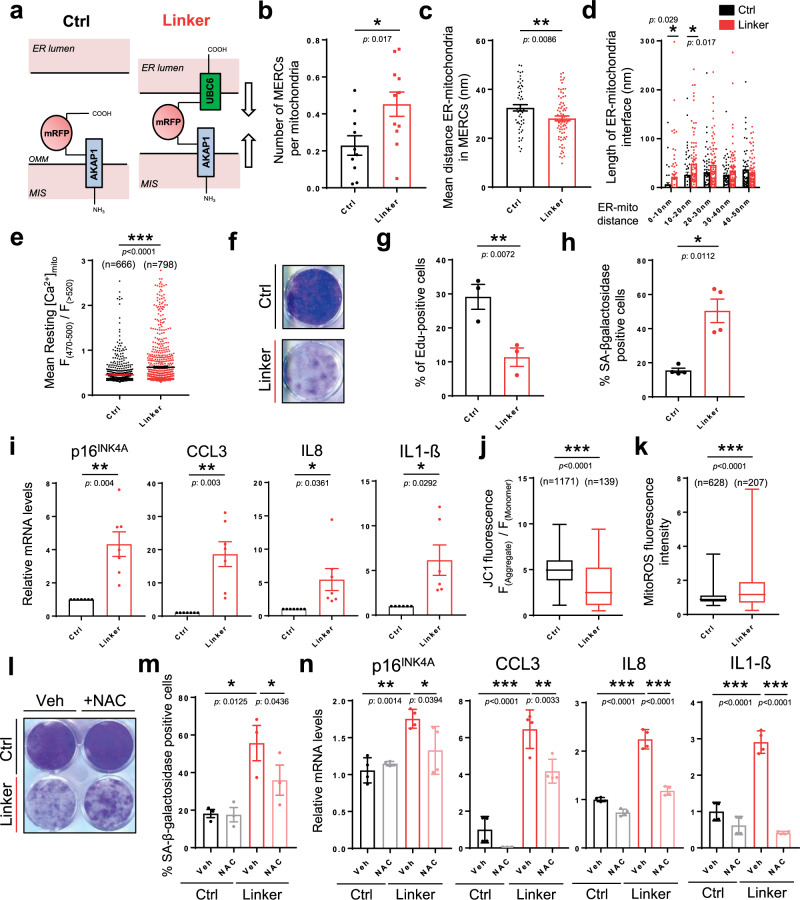
Inducing MERCs promotes premature senescence. **a** Graphical representation of the artificial genetic linker to induce MERCs formation. OMM outer mitochondrial membrane; MIS mitochondrial Intermembrane Space. **b** Using transmission electron microscopy, MERCs number was calculated in Ctrl/Linker-infected MRC5 cells. Mean ± SEM of *n* = 10 (Ctrl) and *n* = 11 (Linker) cells. Unpaired two-tailed Student *t*-test. **c** Mean ER-mitochondria distance in Ctrl/Linker-infected MRC5 cells. Mean ± SEM of *n* = 53 (Ctrl) and *n* = 80 (Linker) MERCs. Unpaired two-tailed Student *t*-test. **d** Mitochondrial membrane length associated with ER, depending on the distance (0-50 nm) between mitochondria and ER in Ctrl/Linker-infected MRC5 cells. Mean ± SEM of *n* = 53 (Ctrl) and *n* = 80 (Linker) MERCs, representative of *n* = 3 independent experiments. Multiple *t*-tests. **e** Steady-state mitochondrial calcium levels in Ctrl/Linker-infected cells. *n*: number of analyzed RFP-positive cells representative of *n* = 3 independent experiments. Mean ± SEM. Two-tailed Mann–Whitney *U* Test. **f** Crystal violet staining for Ctrl/Linker-infected MRC5 cells 12 days after infection, representative of *n* = 3 independent experiments. **g** Quantification of EdU-positive cells for Ctrl/Linker-infected MRC5 cells. Mean ±  SEM of *n* = 3 independent experiments. Paired two-tailed Student *t*-test. **h** Quantification of SA-ß-galactosidase-positive cells in Ctrl/Linker-infected MRC5 cells. Mean ± SEM of *n* = 4 independent experiments. Paired two-tailed Student *t*-test. **i** RT-qPCR representing relative p16^INK4A^, CCL3, IL8, and IL1-ß mRNA levels in Ctrl/Linker-infected MRC5 cells. Mean ± SEM of *n* = 7 independent experiments. Unpaired two-tailed Welch’s *t*-test. **j** Single-cell analysis on RFP-positive Ctrl/Linker-infected cells of mitochondrial membrane depolarisation using JC1 probe. n: number of analyzed cells, representative of *n* = 3 independent experiments. Box plots represent the first quartile, median, and third quartile, whiskers corresponding to min/max values. Two-tailed Mann–Whitney *U* Test. **k** Single-cell analysis of RFP-positive Ctrl/Linker-infected cells of mitochondrial ROS fluorescence. *n*: number of analyzed cells, representative of *n* = 3 independent experiments. Box plots represent the first quartile, median, and third quartile, whiskers corresponding to min/max values. Two-tailed Mann–Whitney *U* Test. **l** Crystal violet staining for Ctrl/Linker-infected MRC5 cells treated with vehicle (Veh) or NAC, representative of *n* = 3 independent experiments. **m** Quantification of SA-ß-galactosidase-positive cells in Ctrl/Linker-infected MRC5 cells, treated with Veh or NAC. Mean ± SEM of *n* = 3 independent experiments. Two-way ANOVA. Paired Tukey’s multiple comparisons test. **n** RT-qPCR representing relative p16^INK4A^, CCL3, IL8, and IL1-ß mRNA levels in Ctrl/Linker-infected MRC5 cells treated with Veh or NAC. Mean ± SD, representative of *n* = 3 independent experiments. Two-way ANOVA. Tukey’s multiple comparisons test. * *p* < 0.05; **p < 0.01; ***p < 0.001.

Loss of *Itpr2* decreases cellular senescence and this effect is correlated with a lower number of MERCs raising the possibility that MERCs directly promote cellular senescence. In order to test this hypothesis, we used a synthetic linker containing a domain anchored to the ER membrane and another one anchored to the mitochondrial outer membrane, as previously described^36^, to force the interaction between mitochondria and the ER (Fig. 4a). TEM analysis showed that constitutive expression of MERC linkers increased the number of MERCs (Fig. 4b and Supplementary Fig. 4a), increased the total length of the ER-mitochondria interface, and brought mitochondria and ER membranes closer according to distances measured between ER and mitochondria in MERCs, probably also tightening some pre-existing MERCs (Fig. 4c, d and Supplementary Fig. 4b) without increasing the perimeter and the number of mitochondria (Supplementary Fig. 4c). Remarkably, forcing MERCs also reduced resting calcium in the ER while promoting in the meantime its accumulation into the mitochondria (Fig. 4e and Supplementary Fig. 4d). Importantly, increasing MERCs via the constitutive expression of linkers led to premature senescence in cells as shown by their decreased ability to proliferate and incorporate EdU (Fig. 4f, g), an increased proportion of SA-β-Gal-positive cells (Fig. 4h) and an increased expression of p16^INK4A^ and various SASP components, including CCL3, IL8 and IL1-β (Fig. 4i).

Mechanistically, the linker-induced mitochondrial depolarization and mitochondrial ROS accumulation (Fig. 4j, k), which can be induced by increased mitochondrial calcium and also are well-known senescence inducers^10,12,37^. Accordingly, N-Acetyl Cysteine (NAC) antioxidant treatment (Fig. 4l–n) as well as knockdown of p53 (Supplementary Fig. 4e–g), a downstream effector of ROS^38,39^, prevented linker-induced senescence. Activation of the pro-inflammatory arm of the SASP can be NF-κB-dependent^38,40,41^. Knockdown of RelA, a member of the NF-κB family of the transcription factor, did not revert proliferation arrest (Supplementary Fig. 4h, i), yet reverted the induction of IL-8 and IL1-β but not of CCL3 in linker-expressing cells (Supplementary Fig. 4i), indicating that the pro-inflammatory SASP induced by the linker is partly regulated by NF-κB activation.

In conclusion, forcing contacts between ER and mitochondrial membranes is sufficient to trigger premature cellular senescence, involving a mitochondrial ROS/p53 pathway and a partial NF-κB-dependent SASP induction.

## Discussion

In this study, we unraveled a role for the calcium channel ITPR2 in the regulation of lifespan, physiological aging and cellular senescence. During these last few years, the functional link between cellular senescence, age-related alterations and lifespan has been demonstrated by eliminating the senescent cells using genetic or pharmacological tools^3,42^. In our study, we observed that loss of *Itpr2* decreases the level of senescence in vitro and in vivo and improves aging, suggesting that ITPR2 regulates aging by impacting cellular senescence.

We mainly studied the impact of *Itpr2* in old mice by focusing on the liver for several reasons. ITPR2 belongs to a family of proteins comprising two other members, ITPR1 and ITPR3, which are known to exert some redundant activities on cancer sensitivity^43^ or on the regulation of senescence^10^. In the bone, where the loss of *Itpr2* does not have any significant beneficial impact, its mRNA level is less abundant than Itpr1 (Supplementary Fig. 5). Of note, in the liver, Itpr2 is known to be highly expressed compared to the two other Itprs (Supplementary Fig. 5)^25^. Moreover, cellular senescence is involved in age-related liver alterations^30,31^. Consequently, the liver appears to be a perfect candidate organ to examine the impact of *Itpr2* deletion on cellular senescence and organ aging and its associated alterations. In line with this hypothesis, *Itpr2* KO delays age-related features of the liver, including both steatosis and fibrosis. Moreover, decreased circulating AST levels as well as a better response to a metabolic challenge observed in *Itpr2* KO are expected to depend, at least in part, on a better liver function during aging^44^. Altogether, our work unravels that liver ITPR2, while it is the most abundant ITPR in this organ, participates actively to its chronological decline.

Cellular senescence induced by short-term injury stimulates wound healing^45^, protects from fibrosis especially in the liver^46–48^ and enhances stemness and regeneration^49^, whereas long-term accumulation of senescent cells promotes fibrosis and degeneration in various contexts^50^. Accordingly, liver regeneration of young *Itpr2* KO mice after hepatectomy is reduced compared to their control littermates^51^. In this latter study, senescence was not examined but we can speculate that it might contribute to liver regeneration. Interestingly, another in vivo study in the brain reported the beneficial effect of *Itpr2* loss in persistent damage-induced phenotype^52^. Once again, senescence has not been evaluated in this photothrombosis-induced cerebral ischemia, but ischemia injuries are known to induce senescence in other models^53,54^. Overall, ITPR2, as a regulator of cellular senescence, could exert both beneficial effects after short-term injury in young individuals but detrimental effects after chronic injuries such as during aging.

Aside from the role of ITPR2 in cellular senescence in vivo and age-related physiological declines, our work also shed light on its role as a potent regulator of the formation and/or maintenance of MERCs. Of note, in a previous study, we did not observe any changes in the MERCs during oncogene-induced senescence in immortalized human mammary epithelial cells according to co-immunofluorescence experiments^10^. Discrepancies between these results might be due to the difference in the senescence system used or/and to the fact that our current study used more sensitive approaches to evaluate MERCs. *Itpr2* KO mice liver or MEFs display a decreased number of MERCs. This decrease occurred only in senescent MEFs when compared to non-senescent ones. How ITPR2 regulates MERCs remains unclear but it has recently been identified as required to maintain MERC^33^. Indeed, according to Bartok and colleagues, ITPR2 participates in maintaining close contacts^33^. Our results support and extend these observations by using both *Itpr2* KO MEFs and mice, demonstrating also a link between MERCs integrity, cellular senescence and aging. ITPR2 could exert this new structural activity by being a tethering species or/and by promoting tether formation by other factors. For instance, as GRP75 links ER and mitochondria by simultaneously binding to ITPRs and VDACs^13,55^, ITPR2 loss might destabilize this ER-mitochondrial tethering system. Taken together, our results and the recent data from literature support that ITPR2 regulates senescence by its canonical calcium channel function and by its non-canonical structural function promoting MERC.

Interestingly, ITPR1, which can regulate senescence similarly to ITPR2^10^, and the linker, which induces MERCs, have also been shown to promote steatosis and to alter glucose homeostasis in obese mice^56^, reinforcing our current observations in *Itpr2* KO mice during aging. Obesity is known to promote cellular senescence, which mediates organismal dysfunctions during obesity including steatosis and glucose homeostasis alterations^31,32,57^. Even though cellular senescence was not investigated in the study by Arruda^56^, we can speculate that the effects of ITPR1 and MERCs are mediated, at least in part, by cellular senescence. ITPR1 and ITPR2 may thus promote senescence and subsequent key drivers of age-related defects in the liver, including steatosis and alterations of glucose homeostasis.

Structural changes at the interface between the ER and the mitochondria induced by ITPR2 likely mediate part of the effect of ITPR2 on senescence. Indeed, we have shown that inducing MERCs by constitutively expressing a synthetic linker leads to premature senescence. MERCs are known to be involved in multiple cellular signaling processes, including calcium transfer to the mitochondria^13,14,34,58^. Mitochondrial calcium contributes to mitochondrial bioenergetics regulation^59^, and its rise can contribute to cellular senescence by inducing ROS production^9–12^. We observed that forced contacts between mitochondria and ER induce mitochondrial calcium accumulation, mitochondrial ROS accumulation and p53-dependent senescence, in line with our previous results deciphering that ITPR2 regulates senescence in a ROS-dependent manner^9–12^. As an association had been reported between mitochondria, ROS, NF-κB transcription factors and SASP^60,61^, we decided to investigate this relationship. We demonstrated the involvement of the RelA NF-κB transcription factor in regulating some SASP factors during linker-induced senescence, and observed a decrease in NF-κB activity in old *Itpr2* KO liver and MEFs according to GSEA analysis (Supplementary Fig. 6). MERCs also constitute signaling platforms involved in pro-inflammatory responses, notably via both formation and regulation of the inflammasome NLRP3^62,63^. In addition, NLRP3 has been associated with the production of SASP components^64^. Interestingly, *Itpr2* KO liver and MEFs display a dampened inflammatory response whereas inducing MERCs enhances the inflammatory response, as evidenced by Il8 and ccl3 expression. This inflammatory response can promote paracrine senescence and mediate part of the pro-aging effects of cellular senescence^2,3^. We can thus speculate that ITPR2, through higher amounts of functional and closer MERCs, could contribute to cellular senescence and aging by two complementary and synergistic mechanisms, namely (i) the increased mitochondrial calcium level and the subsequent ROS production which can induce a p53-dependent cell cycle arrest and a p53- and NF-κB-dependent SASP, as suggested by our results, and (ii) by other mechanisms, for instance inflammasome activation which also triggers pro-inflammatory SASP production known to mediate autocrine senescence. Beyond MERCs and calcium transfer from the ER to the mitochondria, cytosolic calcium and its signaling could also contribute to the senescence phenotype^9^, in line with the decrease in NFAT signaling observed in *Itpr2* KO liver and MEFs according to GSEA analysis (Supplementary Fig. 7).

Overall our study demonstrates that ITPR2 calcium channel and ER-mitochondria contacts promote cellular senescence and physiological aging. How organelles integrate and communicate signals during cellular senescence is unknown. Our study unveils this exciting field of investigation as it paves the way to future studies investigating the roles of organelles contacts in controlling pathways and actors of cellular senescence and aging.

## Methods

### Cell culture and reagents

MRC5 normal embryonic human fibroblasts (ATCC, Manassas, VA, USA), kidney 293T or 293 GP cells (Clontech, Mountain View, CA, USA) were cultured in Dulbecco′s modified Eagle′s medium (DMEM, Life Technologies, Carlsbad, USA) with GlutaMax and supplemented with 10% FBS (Sigma-Aldrich, Saint-Louis, USA) and 1% penicillin/streptomycin (ThermoFisher Scientific). MEFs were prepared with embryos at E12.5 and cultured in Dulbecco′s modified Eagle′s medium with GlutaMax, 10% FBS, 1% penicillin/streptomycin and 1% of Gibco™ MEM Non-Essential Amino Acids Solution (ThermoFisher Scientific). N-acetyl-cysteine (NAC) (A9165, Sigma-Aldrich) has been used directly after infection at a final concentration of 1 mM, and renewed every two days.

### Vectors, transfection, and infection

Linker and control sequences were described in ref. ^36^ and cloned in the lentiviral vector pLV[Exp]-Hygro-CMV by Vectorbuilder. 293T or 293GP virus-producing cells were transfected using the GeneJuice reagent according to the manufacturer’s recommendations (Merck Millipore). Two days after transfection, the viral supernatant was harvested, combined with fresh medium (1/20 for 293T cells 1/2 for 293GP cells) and hexadimethrine bromide (8 μg/mL; Sigma-Aldrich), and used to infect targeted cells. One day later, infected cells were selected with Hygromycin B (ThermoFisher Scientific) at 15 µg/mL or Neomycin (ThermoFisher Scientific) at 75 µg/mL.

### siRNA transfection

MRC5 cells were infected (Ctrl/Linker) and hygromycin-selected for 6 days. They were then seeded and transfected with small interference (si) RNA against RelA or p53 (Dharmacon) previously incubated for 20 min with Dharmafect (Dharmacon) 0.6% in antibiotics and serum-free DMEM with Glutamax (final siRNA concentration in the well: 15 nM). The day after transfection, the medium was changed with DMEM with Glutamax (10% FBS and 1% antibiotics).

### Calcium imaging

For specific ER and mitochondrial ratiometric genetic reporter, MEFs or MRC5 were infected with pLNCX2 G-CEPIA1-ER^65^ or pLNCX2-mito-GEM-GECO1^66^ and neomycin-selected for 10 days. For MRC5, cells were then infected by Linker, hygromycin-selected for 6 days, and seeded in Lab-Tek Chambered Coverglass (ThermoFisher Scientific). Two days after, cells were washed with phenol-free HBSS with Ca^2+^, Mg^2+^ and observed at 37 °C and 5% CO_2_ under a Zeiss LSM 780 confocal microscope. The excitation wavelength was monitored at 408 nm and 565 nm. For calcium reporters, detection of fluorescence was monitored for *F*(470–500 nm) (Calcium-bound reporter) and *F*(>520 nm) (Calcium-free reporter). Ratio *F*(470–500 nm)/*F*(>520 nm) was calculated. For experiments involving the Linker, detection of RFP was monitored for *F*(580 ± 30 nm), and only RFP positive cells were analysed. In order to proceed to single-cell analyses, LSM files were converted to Columbus™ software (Perkinelmer) and single-cell measurement of fluorescence intensity was performed.

### RNA extraction, reverse transcription, and real-time quantitative PCR

RNA was extracted with phenol-chloroform using Upzol (Dutscher, Brumath, France). Synthesis of cDNA was performed using Maxima First cDNA Synthesis Kit (ThermoFisher Scientific) from 1 μg of total RNA. Generated cDNA (50 ng/µL) was used as a template for quantitative PCR (qPCR) run, and mixed with primers (200 nM), SYBR™ Green PCR Master Mix (ThermoFisher Scientific) or TaqMan mix (Roche) and Universal Probe Library probes (100 µM) (ThermoFisher Scientific) for the gene of interest. Reactions were performed in triplicate. qPCR analyses were carried out with the FX96 Thermocycler (Biorad, Hercules, USA). Relative mRNA levels were calculated using the Comparative Ct (ΔΔCT) method. mRNA levels of 2 (*Gapdh/Actb*) housekeeping genes were used for normalization. Primers sequences and housekeeping genes used are listed in Supplementary Table 1.

### Senescence associated-β-galactosidase analysis, crystal violet, EdU assay, mitochondrial JC1, and mitochondrial ROS quantification

For SA-β-Galactosidase assay, cells were washed with PBS 1X, fixed for 5 min in 2% formaldehyde/0.2% glutaraldehyde, rinsed twice in PBS 1X, and incubated at 37 °C overnight in SA-β-Galactosidase staining solutions, freshly prepared (40 mM citric acid/Na phosphate buffer, 5 mM K3[Fe(CN)6], 5 mM K4[Fe(CN)6] 3H2O, 150 mM sodium chloride, 2 mM magnesium chloride, 1 mg/mL X-gal in distilled water). For crystal violet assay, cells were washed with PBS 1×, fixed for 15 min in 3.7% formaldehyde and stained with crystal violet. For EdU Assay, Click-iT™ EdU Alexa Fluor™ 488 Imaging Kit was used according to manufacturer’s recommendations (ThermoFisher Scientific). For JC1, JC1-Mitochondrial Membrane Potential Assay Kit (ab113850, Abcam) was used. JC1 monomers and aggregates were both excited at 488 nm. Detection of fluorescence for JC1 monomers and aggregates were performed respectively at 530 and 590 nm. Ratio F(aggregate)/F(monomer) was subsequently evaluated using Columbus™ software (PerkinElmer) at the single-cell level. Cell Meter™ Mitochondrial Hydroxyl Radical Detection Kit (ATT Bioquest) allowed detection of mitochondrial ROS according to manufacturer’s recommendations. Excitation was monitored at 488 nm, and fluorescence emission was measured at 530 ± 30 nm using Columbus™ software (PerkinElmer) at the single-cell level.

### Animals

C57Bl/6 *Itpr2* KO mice were described in and genotyped as explained in ref. ^17^. WT and KO littermates were used for different experiments. Mice were maintained in laminar-flow boxes under standard conditions (standard diet and water *ad libitum*) at 23 °C with 12-h light and 12-h dark cycles, in the specific pathogen-free (SPF) animal facility Anican platform at the Cancer Research Center of Lyon. Experiments were conducted according to animal care guidelines of European Union and French laws. Protocols and sample sizes, determined in order to have enough samples to detect statistical differences, if any, were approved by the French Ministry of Education and Research (APAFIS#734-2015052915081986).

### PLA and electron microscopy for MERC analysis

For PLA, cells were washed with PBS 1×, and fixed for 10 min in 10% Formaldehyde. 1:1 volume of Glycine 1 M was added to the fixation solution and cells were washed with Glycine 100 mM for 15 min. Cells were permeabilized with 0.1% Triton 1X and next incubated overnight at 4 °C with primary antibodies. Primary antibodies and dilutions used are listed in Supplementary Table 2. Cells were washed with PBS-Tween 0.3% and incubated with PLA probes. Ligation and polymerization steps were performed according to manufacturer’s recommendations (Sigma Aldrich). Acquisition of at least 30 fields per condition was performed using Operetta CLS High-Content Analysis System (PerkinElmer). Columbus™ software (Perkinelmer) was used. After evaluating the number of cells (using Hoescht staining), dots were counted by the software and normalized against this number. Each dot on PLA bar chart represents a mean of *n* = 50–70 cells analyzed.

For transmission electron microscopy, 1:1 volume of glutaraldehyde 4% was added to the culture medium and cells were incubated 15 min at 4 °C. Glutaraldehyde/medium was discarded and 1:1 volume of glutaraldehyde 4 %/cacodylate 0.2 M pH 7.4 was added. Cells were then fixed in glutaraldehyde 2%, washed three times for 1 h at 4 °C, post-fixed with 2% OsO4 1 h at 4 °C, and dehydrated with an increasing ethanol gradient. Impregnation was performed with Epon A (50%) plus Epon B (50%) plus DMP30 (1.7%). Inclusion was obtained by polymerisation at 60 °C for 72 hr. Ultrathin sections (~70 nm thick) were cut on a UCT (Leica) ultramicrotome, mounted on 200 mesh copper grids and contrasted with uranyl acetate and lead citrate. Acquisition of 4–10 fields per cell and 10-20 cells per condition was performed with a Jeol 1400JEM (Tokyo, Japan) transmission electron microscope equipped with an Orius 600 camera and Digital Micrograph at CIQLE platform (UCBL-Lyon). MERCs were determined as distance below 50 nm between ER and OMM membranes. Quantification of MERCs number per mitochondria, length of MERCs and mean/minimal distances <50 nm between ER and OMM membranes per MERC was determined using a macro FiJi software kindly provided by G. Hajnoczky^67^. Using this macro, the number of mitochondria per cell was calculated and the perimeter of each mitochondrion forming a MERC contact and associated with ER membrane in TEM micrographs was assessed.

### Immunoblot and immunohistochemistry

For immunoblot experiments, cells were lysed in RIPA buffer. After protein quantification, 30 μg of proteins were loaded and resolved by SDS-PAGE electrophoresis and transferred to nitrocellulose membranes (Bio-Rad). Membranes were blocked with TBS Tween/Milk 5% for 1 h and incubated at 4 °C with primary antibodies overnight. Primary antibodies and dilutions used are listed in Supplementary Table 2. Membranes were then incubated with secondary antibody for 1 h at room temperature. Detection was performed using ECL kit (Amersham).

For immunohistochemistry, organs were collected and snap-frozen in liquid nitrogen for RNA and protein extraction, or fixed in 10% formalin for 24 h and then ethanol 70%, before processing and paraffin embedding. Paraffin-embedded murine tissues were serially sectioned at 3-mm thickness. After deparaffinization and rehydration, the slides were incubated in 3% hydrogen peroxide in distilled water to block endogenous peroxidases. For heat-induced antigen retrieval, tissue sections were boiled in 10 mmol/L citrate buffer pH 6.0 in a microwave oven for 15 min. The slides were incubated for 30 min with low-background” antibody diluent (DAKO Real) and overnight at 4 °C with the primary antibody (listed in Supplementary Table 2) diluted in the low-background antibody diluent (DAKO Real). After rinsing in PBS, the slides were incubated with a biotinylated secondary antibody bound to a streptavidin peroxidase conjugate (Dako E0468) for 1 h at room temperature. Slides were treated with Streptavidin HRP (Vector) and then bound antibody was revealed with the DAB peroxidase substrate kit (Vector). Sections were counterstained with hematoxylin and the slides were finally dehydrated and mounted. At least 1000 cells taken from five independent fields were quantified.

### Transcriptomic analysis

Transcriptome analysis of liver tissue or MEFs derived from WT or *Itpr2* KO mice were performed using Whole Mouse Genome Microarrays 4x44K v2 (Agilent Technologies) and one-color gene expression Agilent workflow. Total RNA was extracted with NucleoSpin® RNA according to the manufacturer’s recommendations (Macherey-Nagel). cRNAs were synthesized and labeled with Cy3 dye starting from 100 ng of total RNA using one-color Low Input Quick Amp Labeling Kit (Agilent Technologies). After quality control validation, 1650 ng of Cy3-labeled cRNAs purified with RNeasy Mini-spin columns (Qiagen) were hybridized on the 4x44K arrays for 17 h at 65 °C. Microarrays were washed and scanned with an Agilent DNA microarray scanner G2565CA (Agilent Technologies). Fluorescence signals were extracted and normalized with Feature Extraction Software Version 10.5.1.1 (Agilent Technologies) and transferred to Genespring GX 12.6 software (Agilent Technologies) for data processing and data mining. Expression data were normalized in Genespring using the 75th percentile method. Microarray probes were filtered using Agilent flag filter to remove probes with raw signal below 20 in all the conditions tested. Transcriptomic analysis on livers was performed from four independent female mice for each genotype and differentially expressed genes between *Itpr2* KO versus WT with fold change cutoffs > or <2 were selected. Transcriptomic analysis on MEFs was performed from three independent mice for each genotype and differentially expressed genes between *Itpr2* KO and WT were selected using moderated *t*-test *p* value < 0.01 with a Benjamani–Hochberg correction and fold change cutoffs > or <2. For data visualization, hierarchical clustering was performed with the Euclidian metric and complete linkage method. The Gene Ontology (GO) tool from GeneSpring software allowed determination of significant statistical enrichment of biological processes based on computation *p*-values described by standard hypergeometric distribution. Gene Set Enrichment Analysis (GSEA) was performed using the GSEA v2.0.13 software using default parameters. All gene set files for this analysis were obtained from GSEA website (www.broadinstitute.org/gsea/). Datasets are available in GEO GSE139982 for MEF analyses and GSE139967 for liver analyses.

### Blood analysis and phenotyping

Before sacrifice, intracardiac harvesting of blood was performed and measurement of blood AST level was evaluated using the Activated Aspartate Aminotransferase assay on the ARCHITECT c 16000 Systems™. After sacrifice spleens from WT or *Itpr2* KO mice were collected aseptically and single-cell suspensions were prepared in DMEM medium (Invitrogen) containing 2 mM glutamine, 100 mg/ml gentamicin, and 6% FCS. Splenocytes were stained for 30 min at 4 °C with the appropriate mixture of mAbs diluted in staining buffer (PBS supplemented with 1% FCS [Life Technologies] and 0.09% NaN3 [Sigma-Aldrich, Saint Quentin-Fallavier, France]). The following Abs (clones) were used: anti-Mouse CD3e BV421 Clone 145-2C11 (Ref 562600); Anti-Mouse CD45 Alexa Fluor700 Clone 30-F11 (Ref 560510); Anti-Mouse CD4 BV605 Clone RM4.5 (Ref 563151); Anti-Mouse CD8 APC-Cy7 Clone 53-6.7 (Ref 557654); Anti-Mouse CD44 FITC Clone IM7 (Ref 553133); Anti-Mouse CD62L PECy7 Clone MEL-14 (Ref 560516) all BD PharMingen) (Supplementary Table 2). CD4+/CD44high and CD4+/CD44low were considered, respectively, as memory and naive CD4 T cells. CD8+/CD44high and CD8+/CD44low were considered respectively as effector/memory and naive CD8 T cells. All analyses were performed on a Becton Dickinson FACS Fortessa LSRII and analyzed with FlowJo software (TreeStar, Asland, OR, USA). An example of the gating strategy is displayed (Supplementary Fig. 8).

### Measurements of bone mineral density and bone mineral content

After sacrifice, femurs were harvested and stored in EtOH 70%. Bone mineral content and bone mineral density were determined by dual-energy X-ray absorptiometry (DXA) using a Lunar PIXImus densitometer (Wipro, GE Healthcare).

### Liver analyses

For Sirius Red staining, livers were collected and fixed in 10% formalin for 24 h and then ethanol 70%, before processing and paraffin embedding. Paraffin-embedded murine tissues were serially sectioned at 3–4-µm thickness, de-waxed and hydrated. Nuclei were stained with Weigert’s haematoxylin for 8 min, and then the slides were washed for 10 min in running tap water. Picro-sirius red (10%) (Cat#365548 and Cat# P6744-1GA, Sigma Aldrich) was used for staining for 1 h. Slides were washed in two baths of acidified water and dehydrated in three baths of 100% ethanol. Slides were cleared in xylene and mounted. Acquisition of at least 5 fields per mice has been performed. Quantification of the stained area was performed using ImageJ software according to its website’s recommendations.

For red oil staining, livers were collected, snap-frozen, frozen-sectioned at 8-µm thicknesses. Sections were fixed in formalin, washed with tap water and rinsed with 6% isopropanol. Slides were stained with freshly prepared Oil Red O (0.5% of CI 26125 (Sigma-Aldrich) in 60% isopropanol) working solution 15 min, and immediately rinsed with 60% isopropanol. Nuclei were stained with alum hematoxylin 5 dips before mounting. Acquisition of at least 5 fields per mice has been performed and red lipid droplets were counted.

### Intraperitoneal glucose tolerance test (IPGTT)

C57Bl/6 *Itpr2* WT and KO male mice at the indicated ages were starved overnight. Blood tail glycemia was first measured at T0 using Abbott Freestyle Papillon Vision glucometer according to the device manufacturer’s recommendations (Abbott). Intraperitoneal glucose injection (2.5 mg/g) was performed and blood tail glycaemia was then measured as indicated.

### Data representation, reproducibility, and statistical analysis

Demographic data were graphed and processed using Statistica software to compute mean and maximum lifespans, and *p* values (log-rank test) for each cohort. Bar charts represent mean ± SD or SEM as indicated in the figure legend. Box plots represent the first quartile, median, and third quartile with whiskers corresponding to min and max values. Each graph related to electron microscopy is representative of *n* = 3 independent experiments. Statistical analyses for groups were performed as indicated in the figure legend. Depending on the size of sampling, d’Agostini & Pearson or Shapiro-Wilk normality tests were used before proceeding to any analyses. Parametric tests were two-tailed, unpaired or paired: Student’s *t* test (equal variance) or Welch’s *t*-test (for non-equal variance). Mann–Whitney *U* Test was performed for non-parametric tests. For multiple comparisons (>2), one- or two- way ANOVA was performed and subsequent paired or unpaired Tukey’s multiple comparisons test. Two-tailed Spearman Rank Correlation test was used for correlation analysis. All the statistical analyses were performed using GraphPad Prism 7 (*P < 0.05; **P < 0.01; ***P < 0.001; ns non-significant).

### Reporting summary

Further information on research design is available in the Nature Research Reporting Summary linked to this article.

## Supplementary information

Supplementary Information

Reporting Summary

## Data Availability

Datasets are available in GEO GSE139982 for MEF analyses and GSE139967 for liver analyses. Source data are provided with this paper. All remaining data will be available from the corresponding author upon reasonable request.

## Source data

Source Data
