# Supplementary material for: Calcium channel ITPR2 and mitochondria–ER contacts promote cellular senescence and aging
Source: Nat Commun. 2021 Feb 1;12:720. doi: 10.1038/s41467-021-20993-z (PMC7851384; doi:10.1038/s41467-021-20993-z)
Supplement: Supplementary file 1 — Supplementary Information [file 41467_2021_20993_MOESM1_ESM.pdf]

SUPPLEMENTARY INFORMATION

Supplementary Figure 1. a Survival curves of male *Itpr2* WT (n=14) or KO (n=17) C57BL/6 mice. Log-rank test (ns: non-significant). **b** Immunophenotyping of the spleens of 23-month-old mice displaying relative number of naïve (CD44low) CD4+ and CD8+ T-cells and the ratio of naïve/memory T-cells in *Itpr2* WT (n=13) and KO (n=10) in male and female mice. Mean +/- SEM. Unpaired two-tailed t-test. **c** Quantification of the relative number of lipid droplets in liver slices stained with Red Oil of *Itpr2* WT (n=4) and KO (n=4) female mice. Mean +/- SEM. Unpaired two-tailed t-test. **d** GSEA showing decreased enrichment in gene set related to fatty acid metabolism of 23-month old females *Itpr2* KO (n=4) compared to WT (n=4) female mice, according to transcriptomic analyses. **e** Cellular Lipid Metabolic Process Gene Ontology (obtained from the genes downregulated in the livers of 23-month-old females *Itpr2* WT (n=4) and KO (n=4) female mice, according to transcriptomic analyses, corrected p-value: 0.0234. **f** Basal blood glycaemia of young (14-month-old) and old (26-month-old) *Itpr2* WT (n=6) and KO (n=8) male mice. Paired two-tailed t-test (*$p<0.05$; ns: non-significant). **g** Quantification of bone mineral density and content of 23-month old *Itpr2* WT (n=17) and KO (n=16) male and female mice. Mean +/- SEM. Unpaired two-tailed t-test (ns: non-significant). **h** Body weight monitoring during aging of female *Itpr2* WT (n=9) and KO (n=11) mice and male *Itpr2* WT (n=14) and KO (n=17) mice. Mean +/- SEM. Unpaired two-tailed t-test. **i** Quantification of macroscopic tumoral lesions in various organs of *Itpr2* WT (n=17, 7 females and 10 males) and KO (n=16, 4 females and 12 males). Fisher’s exact test (ns: non-significant=p>0,999).

Supplementary Figure 2. a GSEA for Inflammatory Response GO in liver of old *Itpr2* KO (n=4) versus WT (n=4) female mice, according to transcriptomic analysis. **b** Ccl3 and p16^{ink4a}

mRNA levels in liver of *Itpr2* WT (n=14) and KO (n=15) female and male mice. Mean +/- SEM. One-way ANOVA. Multiple comparisons test (*p<0.05). **c** Micrographs of SA-ß-galactosidase assay in late passage *Itpr2* WT and KO MEFs, representative of n=3 biological replicates. Scale bar: 20 µM. **d** Crystal violet staining for late passage MEFs *Itpr2* WT and KO 5 days after seeding, representative of n=3 independent experiments. **e** GSEA for Inflammatory Response GO in late passaged MEFs *Itpr2* WT (n=3) versus KO (n=3), according to transcriptomic analyses. **f-g** MEFs were infected with pLNCX2 G-CEPIA1-ER or pLNCX2-mito-GEM-GECO1 retroviral vector and selected. Single-cell analysis of mitochondrial and ER calcium of late passage *Itpr2* WT and KO MEFs, n: number of analyzed cells. Box plot represents the first quartile, median, and third quartile with whiskers corresponding to min and max values. Two-tailed Mann-Whitney U Test (**p<0.01; ***p<0,001). **h** Single-cell analysis of mitochondrial membrane depolarization using JC1 probe, n: number of analyzed cells. Box plot represents the first quartile, median, and third quartile with whiskers corresponding to min and max values. Two-tailed Mann-Whitney U Test (***p<0,001). **i** Single-cell analysis of mitochondrial ROS fluorescence in late passage *Itpr2* WT and KO MEFs. Red line indicates mean, n: number of analyzed cells. Two-tailed Mann-Whitney U Test (***p<0,0001).

Supplementary Figure 3. a Quantification of PLA using VDAC1 and ITPR1 antibodies in the livers of 23-month-old *Itpr2* WT (n=4) and KO (n=4) male mice. Mean +/- SEM. Unpaired two-tailed t-test (*p<0.05). **b** *Itpr1* and *Vdac1* mRNA levels in livers of 23-month-old *Itpr2* WT (n=4) and KO (n=4) female mice. Mean +/- SEM. Unpaired two-tailed t-test (ns: non-significant). **c** Itpr1, Itpr2, Vdac1 and tubulin protein levels in livers of 23-month-old *Itpr2* WT (n=4) and KO (n=4) female mice. **d-e** Linear regression analyses between the number of MERCs and $p16^{ink4a}$ mRNA levels in the livers or relative intrahepatic fibrosis in 23-month-old *Itpr2* WT (n=8) and KO (n=8) male and female mice. Two-tailed Spearman Rank Correlation

test. **f** *Itpr1* and *Vdac1* mRNA levels in early/late passaged *Itpr2* WT (n=4) and KO (n=4) MEFs. Mean +/- SEM. One-way ANOVA. Multiple comparisons test (ns: non-significant). **g** Itpr1, Itpr2, Vdac1 and tubulin protein levels in early/late passage *Itpr2* WT (n=4) and KO (n=4) MEFs. **h** Representative electron micrographs of late passage *Itpr2* WT and KO MEFs. Magnification X 40 000. ER and mitochondria are artificially labelled respectively in blue and orange. **i** Minimal distance in MERCs of late passage *Itpr2* WT and KO MEFs. Mean +/- SEM of a least 20 cells, n representing individual MERCs of *Itpr2* WT (n=146) and KO (n=138) MEFs. Two-tailed Mann-Whitney U Test (**p<0.01). **j** Number of mitochondria and perimeters of each mitochondrion associated to a MERC in late passage *Itpr2* WT and KO MEFs. For number of mitochondria, mean +/- SEM of n=20 cells (WT and KO). For perimeters, mean +/- SEM of n=114 (WT) and n=123 (KO) mitochondria. Two-tailed Mann-Whitney U Test (**p<0.01; ns: non-significant).

Supplementary Figure 4. a Representative electron micrographs of Ctrl and Linker-infected MRC5 cells. ER and mitochondria are artificially labelled respectively in blue and orange. Arrows indicate ER-mitochondria contacts. **b** Minimal ER-mitochondria distance in MERCs and total length of MERCs in Ctrl and Linker-infected MRC5 cells. Mean +/- SEM of n=53 (Ctrl) and n=80 (Linker) MERCs. Two-tailed Mann-Whitney U Test (**p<0.01; ***p<0.001). **c** Number of mitochondria per cell and perimeters of each mitochondrion associated to a MERC in Ctrl- and Linker-infected MRC5 cells. For number of mitochondria, mean +/- SEM of n=10 (Ctrl) and n=11 (Linker) cells. For perimeters, mean +/- SEM of n=44 (Ctrl) and n=69 (Linker) mitochondria. Two-tailed Mann-Whitney U Test (ns: non-significant). **d** MRC5 cells were infected with pLNCX2 G-CEPIA1-ER calcium reporter encoding retroviral vector, neomycin selected, infected with Ctrl-or Linker-encoding lentiviral vector and next hygromycin selected. Single-cell analysis of ER calcium on RFP positive cells was performed, n: number of analyzed

cells. Two-tailed Mann-Whitney U Test (****p*<0.001). **e** Ctrl or Linker-infected and selected MRC5 cells were transfected with control siRNA or siRNA against p53. Crystal violet staining was performed 12 days after siRNA transfection and seeding of the same number of cells. **f** Quantification of SA-ß-galactosidase-positive cells in Ctrl/Linker-infected MRC5 cells, transfected with siCtrl or sip53. Mean +/- SEM of n=4 independent experiments. Two-way ANOVA. Paired Tukey's multiple comparisons test (**p*<0,05; ***p*<0.001). **g** RT-qPCR representing relative p53, $p16^{INK4A}$, CCL3, IL8 and IL1-ß levels in Ctrl/Linker-infected and selected MRC5 cells transfected with siCtrl or sip53. Mean +/- SEM (n=3-4 independent experiments). Two-way ANOVA. Tukey's multiple comparisons test (**p*<0,05; ***p*<0.01; ****p*<0,001). **h** Crystal violet staining of Ctrl or Linker-infected and selected MRC5 cells, transfected with siCtrl or siRelA. Staining was performed 8 days after siRNA transfection. **i** RT-qPCR representing relative RelA, $p16^{INK4A}$, CCL3, IL8 and IL1-ß levels in Ctrl or Linker-infected and selected MRC5 cells next transfected with siCtrl or siRelA. Mean +/- SEM (n=4 independent experiments for RelA, CCL3, IL-8 or n=3 independent experiments for $p16^{INK4A}$, IL1-ß. Two-way ANOVA. Tukey's multiple comparisons test (**p*<0,05; ***p*<0.01; ****p*<0,001).

Supplementary Figure 5. *Itpr1*, *Itpr2* and *Itpr3* mRNA levels in bone (n=4) and liver (n=6) of *Itpr2* WT female and male mice. One-way ANOVA. Multiple paired t-tests (**p*<0,05; ***p*<0.01; ****p*<0,001).

Supplementary Figure 6. GSEA showing decreased enrichment in gene set related to NF-κB pathway in both late passage MEFs *Itpr2* KO (n=3), compared to WT (n=3) (upper panel), and

liver of *Itpr2* KO (n=4) compared to WT (n=4) female mice (lower panel), according to transcriptomic analyses.

Supplementary Figure 7. GSEA showing decreased enrichment in gene set related to NFAT pathway in both late passage MEFs *Itpr2* KO (n=3), compared to WT (n=3) (upper panel), and liver of *Itpr2* KO (n=4) compared to WT (n=4) female mice (lower panel), according to transcriptomic analyses.

Supplementary Figure 8. FACs gating strategy – representative example. Gating strategy for immunophenotyping of spleen cells. Cell populations were analyzed by gating on live leucocytes and single cell fractions. At least 50,000 leucocytes counts were recorded. A representative example of flow cytometry data (dot plots) is provided here.

Supplementary Figure 1

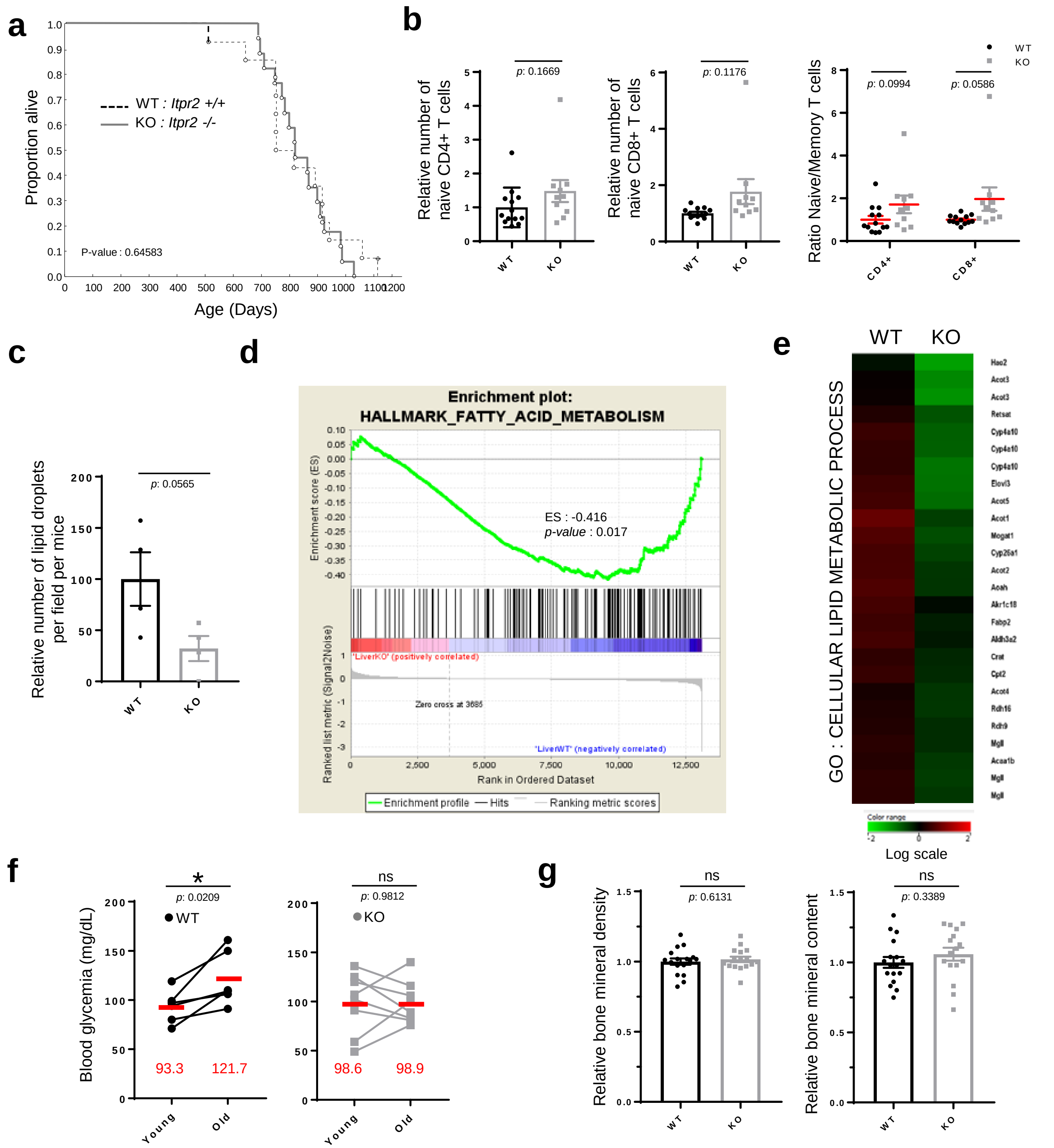

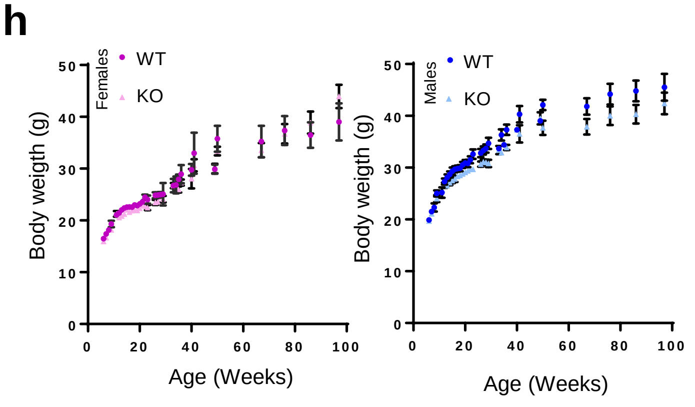

i

	% of mice presenting spontaneous macroscopic lesions at sacrifice						
	WT : Itpr2 +/+			*KO : Itpr2 -/-*			
	**Total**	♀ (n=7)	♂ (n=10)	**Total**	♀ (n=4)	♂ (n=12)	
Lung	**5.88**	0.00	10.00	**0.00**	0.00	0.00	ns
Liver	**5.88**	14.29	0.00	**6.25**	25.00	0.00	ns
Pancreas	**11.76**	14.29	10.00	**6.25**	25.00	0.00	ns
Intestine	**35.29**	42.86	30.00	**37.50**	50.00	33.33	ns
Spleen	**5.88**	14.29	0.00	**6.25**	25.00	0.00	ns
Lymph nodes	**5.88**	14.29	0.00	**6.25**	25.00	0.00	ns

Supplementary Figure 2

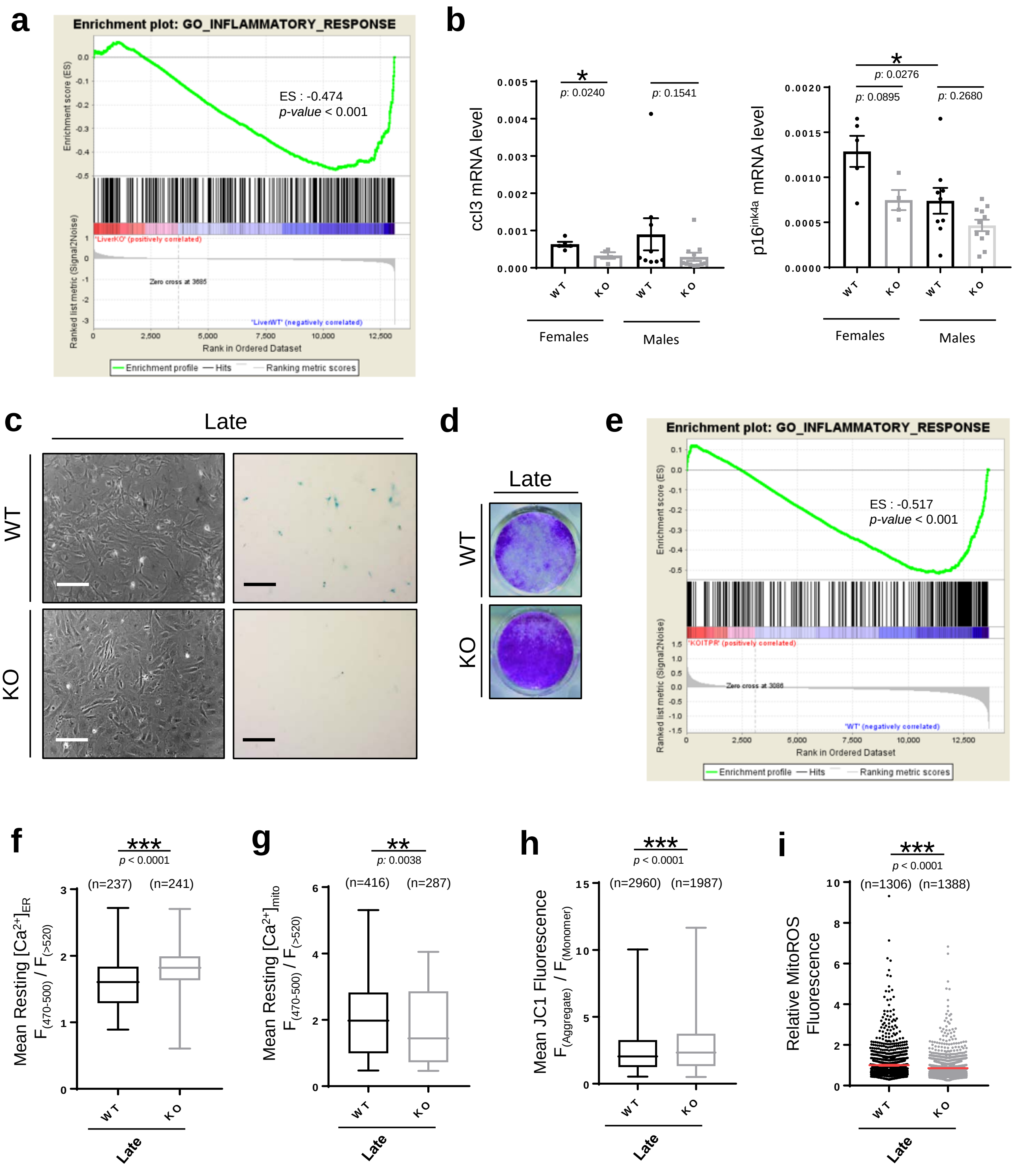

Supplementary Figure 3

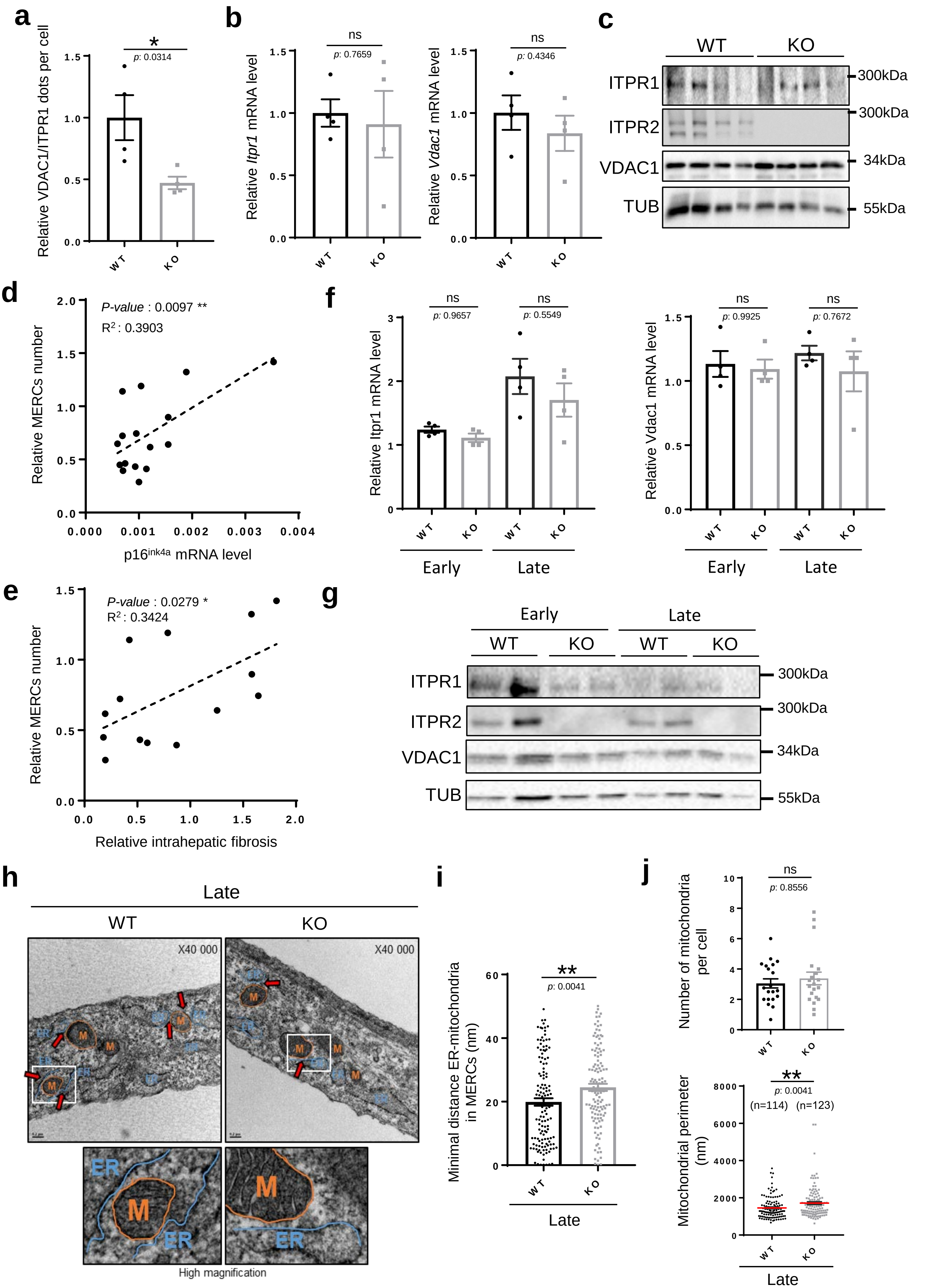

Supplementary Figure 4

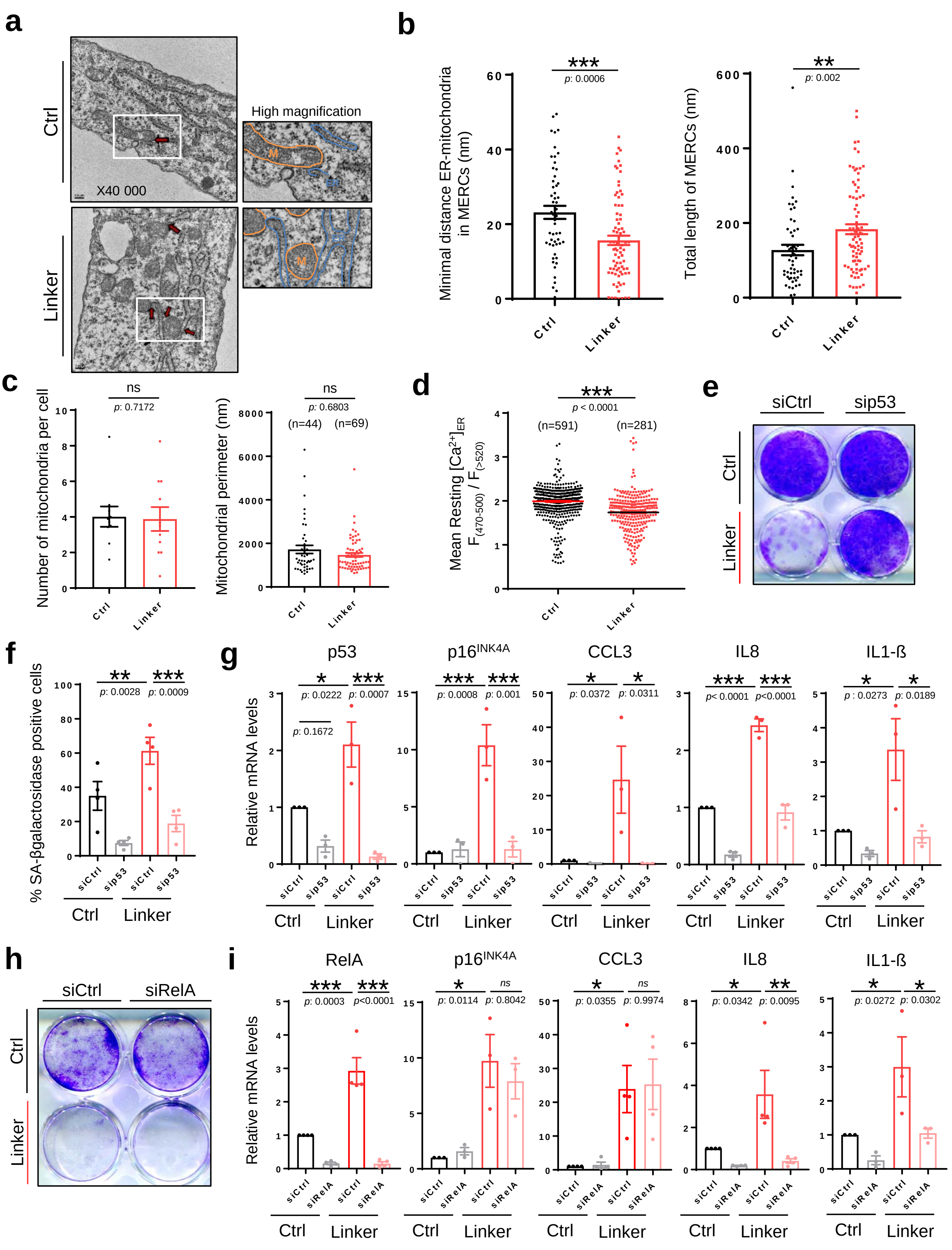

Supplementary Figure 5

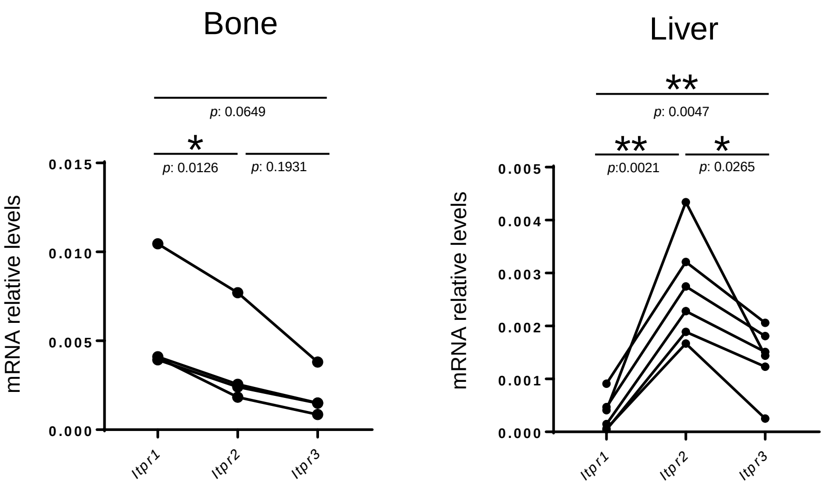

Supplementary Figure 6

MEF

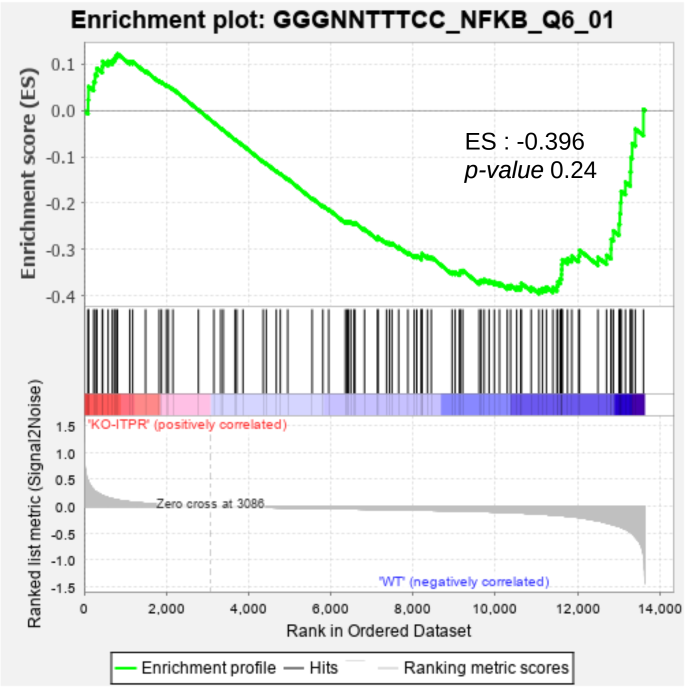

Liver

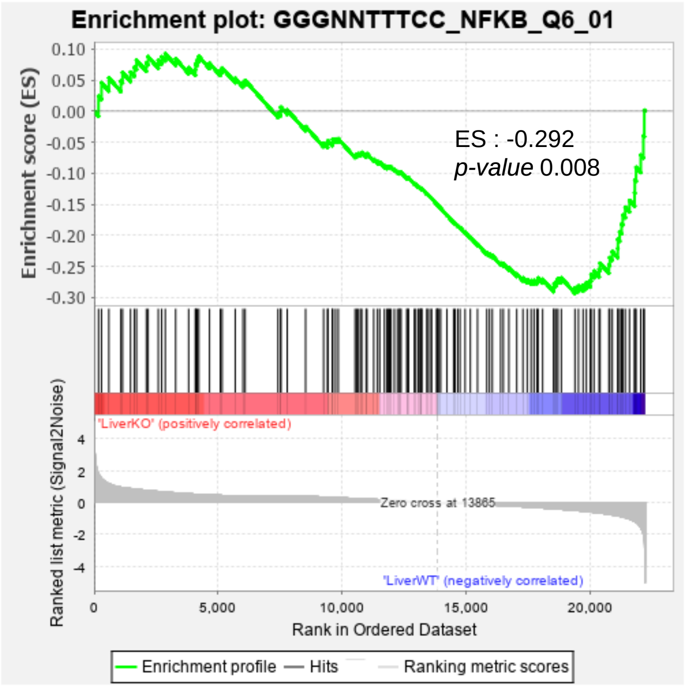

Supplementary Figure 7

MEF

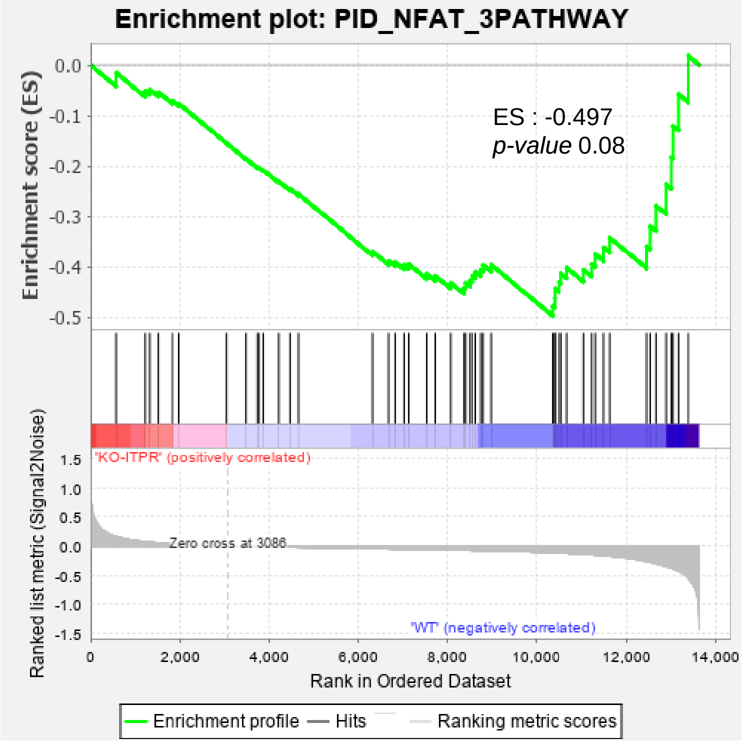

Liver

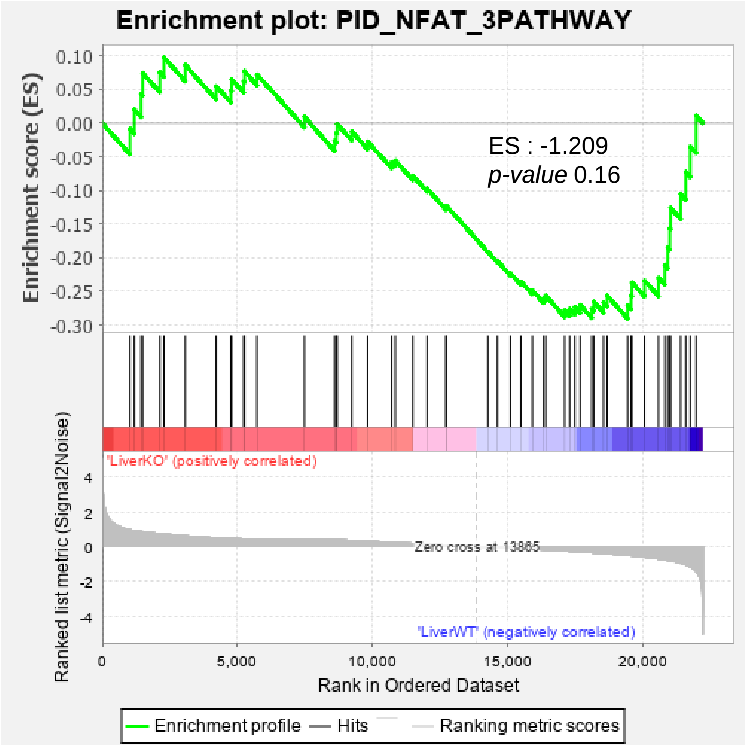

Supplementary Figure 8

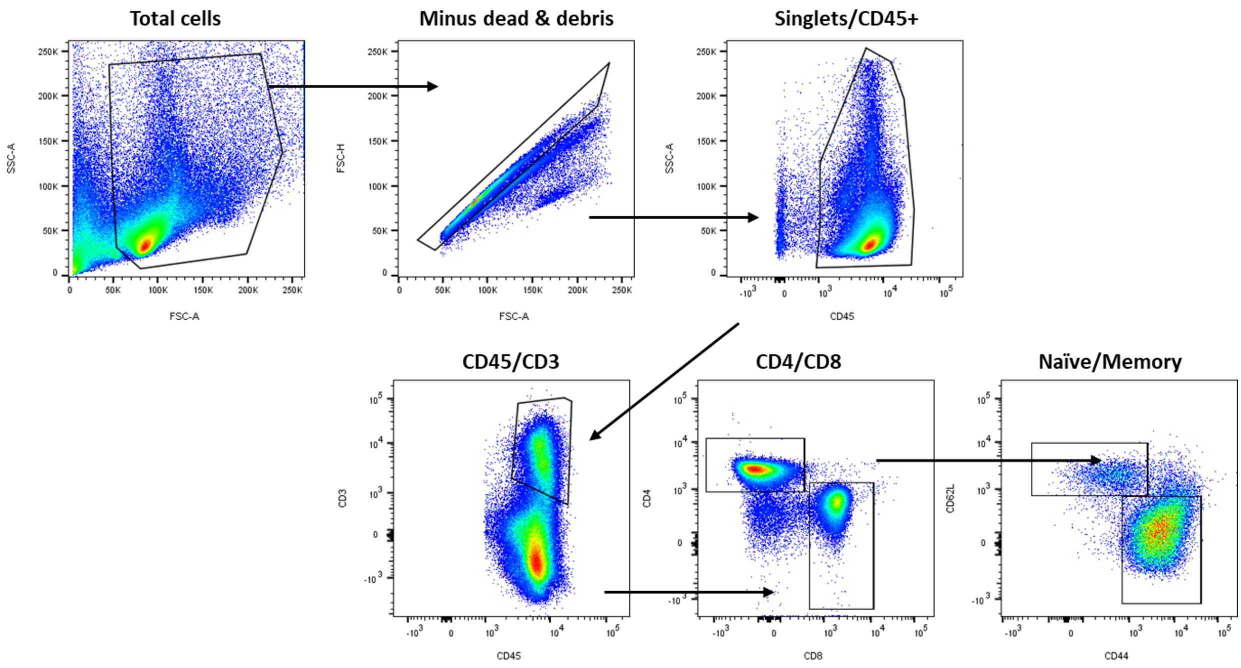

Supplementary Table 1

Gene	Sequence and UPL probes	
**Mouse - UPL**	**Sequence**	**UPL**
Gapdh-Forward	CTAAGGCCAACCGTGAAAAG	#80
Gapdh-Reverse	ACCAGAGGCATACAGGGACA	
ccl3-Forward	TGCCCTTGCTGTTCTTCTCT	#40
ccl3-Reverse	GTGGAATCTTCCGGCTGTAG	
Itpr1-Forward	ACCCTGAGGAAGGTTCTGC	#85
Itpr1-Reverse	GAAGGCATCTTTGGAGGAA	
Itpr2-Forward	TGGTGATGGGAGACCAAACTG	#16
Itpr2-Reverse	GAAGATGTGCCGCATGGT	
Itpr3-Forward	TGGTGATGGAGAACCAAACTG	#80
Itpr3-Reverse	TCTAAGCGTACGTTCAGGATGA	
Vdac1-Forward	ACCTTTGATTCGTCATTCTCG	#48
Vdac1-Reverse	TGCTCCCTCTTGTACCCTGT	
**Mouse - SYBR**		
Actb-Forward	GCCTCCTTCTTGGGTATGG	
Actb-Reverse	AGGTCTTTACGGATGTCAACG	
p16ink4a-Forward	GTGTGCATGACGTGCGGG	
p16ink4a-Reverse	GCAGTTCGAATCTGCACCGTAG	
**Human - UPL**	**Sequence**	**UPL**
ACTB-Forward	ATTGGCAATGAGCGGTTC	#11
ACTB-Reverse	GGATGCCACAGGACTCCAT	
CCL3-Forward	CAGAATCATGCAGGTCTCCAC	#56
CCL3-Reverse	GCGTGTCAGCAGCAAGTG	
IL8-Forward	AGACAGCAGAGCACACAAGC	#72
IL8-Reverse	ATGGTTCCTTCCGGTGGT	
IL1-Beta-Forward	TACCTGTCCTGCGTGTTGAA	#78
IL-1Beta-Reverse	TCTTTGGGTAATTTTTGGGATCT	
RELA-Forward	CTGGCTTGGGGACAGAAG	#39
RELA-Reverse	TCATGAAGAAGAGTCCTTTCAGC	
TP53-Forward	AGGCCTTGGAACTCAAGGAT	#12
TP53- Reverse	CCCTTTTTGGACTTCAGGTG	
**Human - SYBR**		
**ACTB-Forward**	CATGTACGTTGCTATCCAGGC	
**ACTB-Reverse**	CTCCTTAATGTCACGCACGAT	
**p16^{INK4A}-Forward**	CGGTCGGAGGCCGATCCAG	
**p16^{INK4A}-Reverse**	GCGCCGTGGAGCAGCAGCAGCT	

Supplementary Table 2

Protein	Reference	Use	Dilution
$p16^{ink4a}$	sc-1207	IHC	1/1000
	sc-468 (batch K0608)	WB	1/500
ITPR2	sc-398434 (Santa Cruz)	WB	1/500
Tub	T6199 (Sigma-Aldrich)	WB	1/5000
ITPR1	sc-28614 (Santa Cruz)	PLA	1/1000
	sc-271197 (Santa Cruz)	WB	1/500
VDAC1	ab-14734 (Abcam)	PLA	1/1000
		WB	1/500
CD3e	562600 (BD PharMingen)	Flow Cytometry	1/100
CD45	560510 (BD PharMingen)	Flow Cytometry	1/200
CD4	563151 (BD PharMingen)	Flow Cytometry	1/50
CD8	557654 (BD PharMingen)	Flow Cytometry	1/200
CD44	553133 (BD PharMingen)	Flow Cytometry	1/200
CD62L	560516 (BD PharMingen)	Flow Cytometry	1/400
