# Supplementary material for: Calcium channel ITPR2 and mitochondria–ER contacts promote cellular senescence and aging
Source: Nat Commun. 2021 Feb 1;12:720. doi: 10.1038/s41467-021-20993-z (PMC7851384; doi:10.1038/s41467-021-20993-z)
Supplement: Supplementary file 2 — Reporting Summary [file 41467_2021_20993_MOESM2_ESM.pdf]

nature research

Corresponding author(s): David BERNARD

Last updated by author(s): Nov 11, 2020

Reporting Summary

Nature Research wishes to improve the reproducibility of the work that we publish. This form provides structure for consistency and transparency in reporting. For further information on Nature Research policies, see Authors & Referees and the Editorial Policy Checklist .

Statistics

For all statistical analyses, confirm that the following items are present in the figure legend, table legend, main text, or Methods section.

n/a	Confirmed	
☐	☒	The exact sample size (*n*) for each experimental group/condition, given as a discrete number and unit of measurement
☐	☒	A statement on whether measurements were taken from distinct samples or whether the same sample was measured repeatedly
☐	☒	The statistical test(s) used AND whether they are one- or two-sided<br>*Only common tests should be described solely by name; describe more complex techniques in the Methods section.*
☐	☒	A description of all covariates tested
☐	☒	A description of any assumptions or corrections, such as tests of normality and adjustment for multiple comparisons
☐	☒	A full description of the statistical parameters including central tendency (e.g. means) or other basic estimates (e.g. regression coefficient) AND variation (e.g. standard deviation) or associated estimates of uncertainty (e.g. confidence intervals)
☐	☒	For null hypothesis testing, the test statistic (e.g. *F*, *t*, *r*) with confidence intervals, effect sizes, degrees of freedom and *P* value noted<br>*Give P values as exact values whenever suitable.*
☒	☐	For Bayesian analysis, information on the choice of priors and Markov chain Monte Carlo settings
☐	☒	For hierarchical and complex designs, identification of the appropriate level for tests and full reporting of outcomes
☐	☒	Estimates of effect sizes (e.g. Cohen's *d*, Pearson's *r*), indicating how they were calculated

Our web collection on statistics for biologists contains articles on many of the points above.

Software and code

Policy information about availability of computer code

Data collection	none
Data analysis	For calcium imaging: In order to proceed to single-cell analyses, LSM files were converted to ColumbusTM software (Perkinelmer) and single-cell measurement of fluorescence intensity was performed.<br>For mitochondrial ROS: Fluorescence emission was measured at 530 +/- 30 nm using ColumbusTM software (PerkinElmer) at single-cell level.<br>For JC1 measurements: Detection of fluorescence for JC1 monomers and aggregates were performed respectively at 530nm and 590nm. Ratio F(aggregate)/F(monomer) was subsequently evaluated using ColumbusTM software (PerkinElmer) at single-cell level.<br>For intrahepatic liver fibrosis measurement: ImageJ software was used.<br>For PLA dots measurements: ColumbusTM software (Perkinelmer) was used. After evaluating the number of cells (using Hoescht staining), dots were counted by the software and normalized against this number.<br>For microarray analysis: Gene Set Enrichment Analysis (GSEA) were performed using the GSEA v2.0.13 software using default parameters.

For manuscripts utilizing custom algorithms or software that are central to the research but not yet described in published literature, software must be made available to editors/reviewers. We strongly encourage code deposition in a community repository (e.g. GitHub). See the Nature Research guidelines for submitting code & software for further information.

Data

Policy information about availability of data

All manuscripts must include a data availability statement. This statement should provide the following information, where applicable:

- Accession codes, unique identifiers, or web links for publicly available datasets
- A list of figures that have associated raw data
- A description of any restrictions on data availability

data availability statement is included: Datasets are available in GEO GSE139982 for MEF analyses and GSE139967 for liver analyses. The source data underlying the Figures 1(a,b,c,d,e,f,g), 2(b,c,d,e,f,g,h,i), 3(a, b,c,d,e,f), 4(b,c,d,e,f,h,i,j,k,l,m,n) and Supplementary Figures 1(a,b,c,f,g,h,i), 2(b,f,g,h,i), 3(a,b,c,d,e,f,g,i,j), 4(b,c,d,f,g,i), 5 are provided as a Source Data file. All remaining data will be available from the corresponding author upon reasonable request.

Field-specific reporting

Please select the one below that is the best fit for your research. If you are not sure, read the appropriate sections before making your selection.

☒ Life sciences ☐ Behavioural & social sciences ☐ Ecological, evolutionary & environmental sciences

For a reference copy of the document with all sections, see nature.com/documents/nr-reporting-summary-flat.pdf

Life sciences study design

All studies must disclose on these points even when the disclosure is negative.

Sample size	Experiments were conducted according to animal care guidelines of European Union and French laws. Protocols and sample sizes, determined in order to have enough samples to detect statistical differences, if any, were authorized by the French Ministry of Education and Research (APAFIS#734-2015052915081986). Sample sizes have been determined in order to have enough samples to detect statistical differences, if any.
Data exclusions	none
Replication	In vivo data has been performed on one littermate aging cohort (n indicated on figure legend).<br>For in vitro data, all attempts of replication were successful when internal controls were fine.
Randomization	For animal and MEFs experiments, groups were not randomized and only formed according to the genotypes of the mice (ITPR2+/+ or ITPR2-/-).
Blinding	For immunofluorescence experiments and single-cell experiments, semi-automatic methods were used (using ColumbusTM software (PerkinElmer), for analysis guaranteeing random selection.<br>For mice experiments, an identification code was given to each mice and samples. The corresponding genotype was checked only after performing experiments.

Reporting for specific materials, systems and methods

We require information from authors about some types of materials, experimental systems and methods used in many studies. Here, indicate whether each material, system or method listed is relevant to your study. If you are not sure if a list item applies to your research, read the appropriate section before selecting a response.

Materials & experimental systems

n/a	Involved in the study	
☐	☒	Antibodies
☐	☒	Eukaryotic cell lines
☒	☐	Palaeontology
☐	☒	Animals and other organisms
☒	☐	Human research participants
☒	☐	Clinical data

Methods

n/a	Involved in the study	
☒	☐	ChIP-seq
☐	☒	Flow cytometry
☒	☐	MRI-based neuroimaging

Antibodies

Antibodies used	Antibodies used are listed in Table S2:<br>p16ink4a antibody (sc-1207 for IHC and sc-468 (batch K0608) for WB); ITPR2 antibody (c-398434); Tubulin antibody (T6199 Sigma-Aldrich); ITPR1 antibody (sc-28614 for PLA and sc-271197 for WB); VDAC1 antibody (ab-14734 Abcam); CD3e BV421 antibody (145-2C11 PharMingen); CD45 Alexa Fluor700 antibody (30-F11 PharMingen); CD4 BV605 antibody (RM4.5

	PharMingen); CD8 APC-Cy7 antibody (53-6.7 PharMingen); CD44 FITC antibody (IM7 PharMingen); CD62L PECy7 antibody (MEL-14 PharMingen).<br>Dilution for each antibody and application has been provided in Table S2.
Validation	Figure 2C: p16 IHC, sc-1207, this Ab was broadly validated (251 citations found for this Ab according to CiteAb website) and localization of the staining was as expected in our study; Figure 2I: p16 WB, sc-468, this Ab broadly was broadly validated (168 citations found in CiteAb website) and it was validated in our study according to the size of the detected band : ITPR2 WB, sc-398434, Ab validated by the size of the band detected and the disappearance of the band in Itpr2 KO mice : tubulin WB, T6199, broadly validated (cited more than 1000 times according to CiteAb website) and validated by the size of the detected band ; Figure 3A and 3C: ITPR1 VDAC1 antibodies for PLA, previously validated in different studies of the Dr Jennifer Rieusset, also coauthor of this study; Supplemental Figure 3C and G: ITPR1 antibody for western blot (sc-271197 (Santa Cruz)) was validated according to the size of the detected band and it was used in several articles according to CiteAb website. VDAC1 (ab-14734) was broadly used and cited (cited more 290 times according to CiteAb website) and size of the detected band was as expected. ITPR2 and Tubulin antibodies were already commented.<br><br>For immunophenotyping, antibodies against CD4 (RM4.5), CD8 (53-6.7), CD44 (IM7.8), CD3e (145-2C11), CD45 (30-F11), CD62L (MEL-14) (all BD PharMingen) were all fully validated by the supplier and in previsous analyses of Drs Sophia Djebali and Jacqueline Marvel, both coauthors of this study.<br><br>These antibodies were used only against mouse tissues and derived cells.

Eukaryotic cell lines

Policy information about cell lines

Cell line source(s)	MRC5 : ATCC / 293T + 293 GP Cells : Clontech / MEFs: produced by ourself.
Authentication	Cells were amplified and frozen upon receipt from the company before their use. Mefs were prepared in the lab. No further authentifications were performed
Mycoplasma contamination	Virus producing cells and MRC5 were checked for mycoplasma contamination, MEFs were not. If positive, cells were treated with Plasmocyn for two weeks. Only negative cells were used for experiments presented in the manuscript.
Commonly misidentified lines<br>(See ICLAC register)	No commonly misidentified cells lines were used in this study.

Animals and other organisms

Policy information about studies involving animals; ARRIVE guidelines recommended for reporting animal research

Laboratory animals	C57Bl/6 / Males and Females / Age : anlaysis were performed on 23-month old mice after sacrificed, or in live mice on the aging cohort up to their death or reaching ethical limit.<br>housing conditions:<br>Mice were maintained in laminar-flow boxes under standard conditions (standard diet and water ad libitum) at 23°C with 12 hrs light and 12 hours dark cycles, in the specific pathogen-free (SPF) animal facility Anican platform at the Cancer Research Center of Lyon
Wild animals	This study dit not involve wild animals.
Field-collected samples	No field collected samples were used in the study.
Ethics oversight	Protocols and sample sizes, determined in order to have enough samples to detect statistical differences, if any, were approved by the French Ministry of Education and Research (APAFIS#734-2015052915081986).

Note that full information on the approval of the study protocol must also be provided in the manuscript.

Flow Cytometry

Plots

Confirm that:

- [ ] The axis labels state the marker and fluorochrome used (e.g. CD4-FITC).
- [ ] The axis scales are clearly visible. Include numbers along axes only for bottom left plot of group (a 'group' is an analysis of identical markers).
- [ ] All plots are contour plots with outliers or pseudocolor plots.
- [x] A numerical value for number of cells or percentage (with statistics) is provided.

Methodology

Sample preparation	After sacrifice spleens from WT or Itpr2 KO mice were collected aseptically and single-cell suspensions were prepared in DMEM medium (Invitrogen) containing 2 mM glutamine, 100 mg/ml gentamicin, and 6% FCS. Splenocytes were stained for 30 min at 4° C with the appropriate mixture of mAbs diluted in staining buffer (PBS supplemented with 1% FCS [Life Technologies] and 0.09% NaN3 [Sigma-Aldrich, Saint Quentin-Fallavier, France]).
Instrument	All analyses were performed on a Becton Dickinson FACS Fortessa LSRII.
Software	FlowJo software (TreeStar, Asland, OR, USA) was used.
Cell population abundance	Cell populations were analyzed by gating on live leucocytes and single cell fractions. At least 50,000 leucocytes counts were recorded. A representative example of flow cytometry data (dot plots) is provided in the supplementary figure.
Gating strategy	An example of the gating strategy is displayed (Supplementary Fig. 8).

☒ Tick this box to confirm that a figure exemplifying the gating strategy is provided in the Supplementary Information.
